# Retinal pigment epithelium extracellular vesicles are potent inducers of age‐related macular degeneration disease phenotype in the outer retina

**DOI:** 10.1002/jev2.12295

**Published:** 2022-12-21

**Authors:** Marzena Kurzawa‐Akanbi, Phillip Whitfield, Florence Burté, Pietro Maria Bertelli, Varun Pathak, Mary Doherty, Birthe Hilgen, Lina Gliaudelytė, Mark Platt, Rachel Queen, Jonathan Coxhead, Andrew Porter, Maria Öberg, Daniela Fabrikova, Tracey Davey, Chia Shyan Beh, Maria Georgiou, Joseph Collin, Veronika Boczonadi, Anetta Härtlova, Michael Taggart, Jumana Al‐Aama, Viktor I Korolchuk, Christopher M Morris, Jasenka Guduric‐Fuchs, David H Steel, Reinhold J Medina, Lyle Armstrong, Majlinda Lako

**Affiliations:** ^1^ Biosciences Institute, Faculty of Medical Sciences Newcastle University Newcastle upon Tyne UK; ^2^ Glasgow Polyomics and Institute of Infection, Immunity and Inflammation, College of Medical, Veterinary and Life Sciences University of Glasgow Glasgow UK; ^3^ The Welcome‐Wolfson Institute for Experimental Medicine Queen's University Belfast Belfast UK; ^4^ Lipidomics Research Facility University of the Highlands and Islands Inverness UK; ^5^ Translational and Clinical Research Institute Newcastle University Newcastle upon Tyne UK; ^6^ Loughborough University Loughborough UK; ^7^ Institute of Biomedicine, Department of Microbiology and Immunology, Sahlgrenska Academy University of Gothenburg Gothenburg Sweden; ^8^ Wallenberg Center for Molecular and Translational Medicine University of Gothenburg Gothenburg Sweden; ^9^ Electron Microscopy Research Services Newcastle University Newcastle upon Tyne UK; ^10^ Faculty of Medicine King Abdulaziz University Jeddah Saudi Arabia; ^11^ The Institute of Medical Microbiology and Hygiene University Medical Center Freiburg (Universitätklinikum Freiburg) Freiburg Germany

**Keywords:** age‐related macular degeneration, complement factor H, extracellular vesicles, human induced pluripotent stem cells, retinal pigment epithelium, photoreceptors, retina

## Abstract

Age‐related macular degeneration (AMD) is a leading cause of blindness. Vision loss is caused by the retinal pigment epithelium (RPE) and photoreceptors atrophy and/or retinal and choroidal angiogenesis. Here we use AMD patient‐specific RPE cells with the Complement Factor H Y402H high‐risk polymorphism to perform a comprehensive analysis of extracellular vesicles (EVs), their cargo and role in disease pathology. We show that AMD RPE is characterised by enhanced polarised EV secretion. Multi‐omics analyses demonstrate that AMD RPE EVs carry RNA, proteins and lipids, which mediate key AMD features including oxidative stress, cytoskeletal dysfunction, angiogenesis and drusen accumulation. Moreover, AMD RPE EVs induce amyloid fibril formation, revealing their role in drusen formation. We demonstrate that exposure of control RPE to AMD RPE apical EVs leads to the acquisition of AMD features such as stress vacuoles, cytoskeletal destabilization and abnormalities in the morphology of the nucleus. Retinal organoid treatment with apical AMD RPE EVs leads to disrupted neuroepithelium and the appearance of cytoprotective alpha B crystallin immunopositive cells, with some co‐expressing retinal progenitor cell markers Pax6/Vsx2, suggesting injury‐induced regenerative pathways activation. These findings indicate that AMD RPE EVs are potent inducers of AMD phenotype in the neighbouring RPE and retinal cells.

## 1 INTRODUCTION

Age‐related macular degeneration (AMD) is a degenerative disease of the macular region of the retina, leading to progressive loss of central vision (Mitchell et al., 2018). It is the third leading cause of visual loss globally (World Health Organization), with 8.7% of the worldwide population affected by AMD and nearly 300 million people projected to have the disease by 2040 (Wong et al., 2014), posing a substantial socioeconomic burden. Drusen accumulation and abnormalities in the retinal pigment epithelium (RPE) are the pathognomonic features of AMD. The disease progresses from an asymptomatic early stage, characterised by the presence of medium‐sized drusen (63–125 μm) in the macular region, through intermediate stages with enlarged drusen (>125 μm) and retinal pigmentary changes, to the late stages of either dry (geographic atrophy) or wet (neovascular) AMD (Mitchell et al., 2018). Dry AMD is usually slowly progressive with choriocapillaris and RPE loss which eventually leads to the formation of atrophic patches with accompanying photoreceptor degeneration (Holz et al., 2014), with no treatment available to date. Wet AMD occurs when new blood vessels form under the retina with leakage, haemorrhage and fibrosis causing rapid loss of central vision (Coleman et al., 2008). Anti‐VEGF (vascular endothelial growth factor) therapies are an established treatment for this condition but need to be started early in the course and do not alter the underlying disease process.

AMD is a multifactorial disease with genetic and environmental factors recognised to play a role in disease susceptibility. Fifty‐two common and rare genetic variants at 34 loci have been identified to be associated with AMD (Fritsche et al., 2016), with the complement factor H gene (*CFH*) Y402H polymorphism making a substantial contribution to the disease susceptibility. A large meta‐analysis has shown that *CFH* Y402H confers approximately 2‐fold higher risk of developing late AMD per copy of the risk allele in persons of European ancestry (Sofat et al., 2012). Advanced age, European descent and smoking are strong environmental risk factors for AMD (Khan et al., 2006; Wong et al., 2014), with some, however, inconsistent results for the association between AMD and high‐fat diet, and cardiovascular risk factors such as hypertension and total serum cholesterol.

It is likely that a combination of inflammatory processes (complement pathway dysregulation, inflammasome activation), intrinsic (e.g., photo‐oxidation) and extrinsic (e.g., cigarette smoke) oxidative insult to the retina, age‐related metabolic impairment (mitochondrial, autophagic and endoplasmic reticulum stress), together with the extremely limited proliferative and regenerative capacity of the RPE in situ (Grierson et al., 1994), leads to the RPE cell demise and AMD. Autophagy dysfunction and local inflammatory processes in aged RPE might be ways that the extracellular debris accumulates beneath the RPE. We have recently shown that dysfunctional lysosomal degradation, directly linked to the complement system overactivation occurs in AMD RPE cells (Cerniauskas et al., 2020). AMD drusen are immunopositive for markers of autophagy and extracellular vesicles (EVs), that are part of the RPE cell secretome (Wang et al., 2009). It is likely that EVs, released by the cells as part of a normal process (Klingeborn et al., 2017), accumulate underneath and around the RPE and may be involved in drusen formation. In favour of this hypothesis, accumulations of EVs at the base of photoreceptors outer segments have been observed in a progressive rod‐cone degeneration mouse model (Spencer et al., 2019), resembling pseudo‐drusen accumulation in AMD. Multi‐vesicular structures were also observed on transmission electron microscopy of drusen of human RPE cultured on porous supports, allowing the cells to develop their natural polarisation and secretory functions (Johnson et al., 2011). Despite the observations, the molecular and functional nature of these vesicles secreted by the AMD‐RPE cells remains to be explored.

There is a large body of evidence showing that oxidative stress enhances autophagy (Kroemer et al., 2010). EVs production, as part of the inherent mechanism to maintain cellular homeostasis, may be an alternative way to reduce cell stress through secretion (Baixauli et al., 2014). Interestingly, human ARPE19 cells under low‐level oxidative stress conditions had enhanced EVs secretion, containing VEGF receptor 2 that mediated their pro‐angiogenic properties (Atienzar‐Aroca et al., 2018). Accumulation of EVs around the RPE might therefore be a contributing factor in the progression from dry to wet AMD, and their role in promoting new blood vessel growth has been reported in other conditions (Todorova et al., 2017). Moreover, the role of EVs in inflammatory processes, including complement component transport and complement activation (Karasu et al., 2018) is subject to investigation, and RPE EVs may transport the C3 complement component as shown previously (Wang et al., 2009).

Data from multiple studies suggest that autophagic degradation regulates EVs biogenesis pathways, and both systems work in harmony to complement their potential inefficiencies to minimise cellular stress (Salimi et al., 2020). As such, it has been shown that chemical inhibition of lysosomal degradation with bafilomycin A1 or ammonium chloride leads to an increase in secretion of EVs and EV‐contained alpha‐synuclein in SH‐SY5Y cells, modelling Parkinson's disease pathogenesis (Alvarez‐Erviti et al., 2011). Similarly, in Niemann‐Pick type C1, the disease‐associated accumulation of cholesterol and sphingolipids in endo‐lysosomal compartments correlated with enhanced secretion of cholesterol‐rich EVs and has been suggested to play a role in regulating cellular cholesterol metabolism (Strauss et al., 2010). This evidence suggests that compromised lysosomal degradation or endo‐lysosomal overload with molecules may be a stimulus for the cells to activate waste/unnecessary material removal via secretory routes (Buratta et al., 2020). Consequently, the autophagic and exosomes markers that were found underneath the RPE in old mice retinas and drusen from AMD eyes may be an indicator of compensatory mechanisms in aged RPE and suggestive of EVs contribution towards drusen formation (Wang et al., 2009).

In a very recent study, Flores‐Bellver et al. reported that RPE cells under homeostatic conditions secrete EVs, whose cargo is enriched in proteins involved in oxidative stress, complement activation and inflammation as well as drusen formation (Flores‐Bellver et al., 2021). Although the authors observed an increase in drusen associated proteins in EV cargo in response to cigarette smoke extract, they did not extend these studies to patient‐specific RPE cells, harbouring genetic risk factors, which show a clear causal association with AMD risk. To fully understand the role of EVs in the pathobiology of AMD, we focus herein on the molecular and functional characterisation of EVs secreted by the AMD‐patient derived induced pluripotent stem cell (iPSC) RPE cells with the common homozygous polymorphism in the complement factor H gene (*CFH* Y402H) and their role in disease signalling in the outer retina and AMD progression.

## 2 MATERIALS AND METHODS

### 2.1 Cell cultures

#### 2.1.1 Induced pluripotent stem cells (iPSC)

iPSCs were generated from dermal fibroblasts from two patients with wet AMD associated with the homozygous *CFH* Y402H polymorphism (high‐risk AMD RPE) and two age matched donors with no clinical or genotypic indication of ocular disease (low‐risk control RPE), as reported previously (Hallam et al., 2017). Two additional control cell lines (WT1 and WT3) were used to confirm the reproducibility of our iPSC and iPSC‐RPE culture (Buskin et al., 2018). iPSCs were cultured in mTeSR1 (Stem Cell Technologies) growth media on Matrigel Growth Factor Reduced Basement Membrane Matrix (Corning) in humidified, 5% CO_2_, 37°C conditions. iPSCs were passaged every 4–6 days using Versene (EDTA) 0.02% (Lonza).

#### 2.1.2 iPSC‐RPE

For differentiation into RPE cells, iPSCs were brought to 95%–100% confluence, maintained for additional 1–2 days and fed with RPE differentiation media (Advanced RPMI 1640, 10% KnockOut serum replacement, 2% B27 supplement, 1% GlutaMAX and 1% Penicillin‐Streptomycin (all ThermoFisher)). Media was partially replaced daily. Once pigmented RPE cell patches appeared (2‐3 weeks), media was partially replaced three times a week, and once established—twice a week. RPE patches were excised with a scalpel, dissociated with TrypLE Select Enzyme (10×) (ThermoFisher), sieved through a 100 μm cell strainer and replated onto Matrigel coated 24‐well plates (125,000 cells/cm^2^). Once showing pigmentation and hexagonal morphology, RPE cells were expanded by passaging at a 1:2 to 1:3 ratio using TrypLE Select Enzyme (10×). For assays, RPE cells were seeded onto Matrigel coated 0.4 μm PET hanging cell culture inserts (Merck) at a density of 450,000 cells/cm^2^. Subsequent cell passages were used to generate replicates for the analyses.

#### 2.1.3 Retinal organoids

For retinal organoid differentiation, control iPSCs were brought to 90% confluence and dissociated with Accutase (Thermo Fisher Scientific). Cells were seeded at a density of 7000 cells per well in 96‐well Lipidure coated plates (Amsbio) in 100 μl of mTeSR1 (Stem Cell Technologies) with 10 μM ROCK inhibitor (Y27632, Tocris). After 48 h, 200 μl of differentiation medium was added (DMEM/F12 containing 20% KnockOut serum replacement, 2% B‐27 Supplement, 1% Pen/Strep, 1% Non‐Essential Amino Acids (NEEA; all Thermo Fisher Scientific) and 5 ng/ml IGF‐1 (Sigma Aldrich)). Half of the medium was replaced three times a week. On day 18 of differentiation retinal organoids were transferred to low attachment 6‐well plates (Costar, Corning) at a density of 24 organoids per well and the medium was changed to DMEM/F12 containing 10% Fetal Bovine Serum (FBS; Life Technologies), 2% B‐27 Supplement, 1% Pen/Strep, 1% NEEA, 5 ng/ml IGF‐1 and 0.1 mM Taurine (Sigma Aldrich). The medium was changed three times a week. The medium composition changed from day 30 of differentiation onwards to DMEM/F12 containing 10% FBS, 2% B‐27 Supplement, 1% Pen/Strep, 1% NEEA, 1% N‐2 Supplement, 10 ng/ml IGF‐1, 1% Chemically Defined Lipid Concentrate (all Thermo Fisher Scientific), 0.1 mM Taurine and T3 (40 ng/ml; Sigma Aldrich) and retinal organoids were further cultured until day 221 of differentiation. Retinoic Acid (RA; 1 μM; Sigma‐Aldrich) was added from day 90 to day 120 of differentiation.

### 2.2 EVs isolation

For EVs isolation cells were washed with exosome free medium (Advanced RPMI 1640, 1% exosome depleted Fetal Bovine Serum, 2% serum free B27 supplement, 1% GlutaMAX and 1% Penicillin‐Streptomycin (all ThermoFisher)) and maintained in the medium. Alternatively, medium without B27 supplement was used, as this was found to not adversely affect the mature RPE cells viability. Medium (with B27 supplement) was tested by Tunable Resistive Pulse Sensing (TRPS), with no particles above 10^6^/ml detected. Collection of cell conditioned media (CCM) was performed every 48–72 h. For ‘omics’ analyses, at least 250 ml of CCM per sample was used. To isolate EVs, CCM was pre‐clarified by centrifugation at 3000 × *g* for 10 min and concentrated to 500 μl using Amicon 15 10 kDa or 30 kDa MWCO (Merck). The concentrate was applied onto the qEV original 70 nm size exclusion chromatography column (SEC, Izon) and EVs were eluted in Phosphate Buffered Saline (PBS, Sigma) in fractions 7, 8 and 9 (1.5 ml after the void volume) as indicated by the manufacturer. The EV suspension was finally concentrated using the Amicon 15 3 kDa MWCO ultrafiltration units (Merck) (200‐400 μl final volume equal for all samples per assay). For the proteomics analysis, SEC‐purified EVs in PBS were ultracentrifuged at 120,000 × *g* for 2 h at 4°C in a Beckman Coulter ultracentrifuge using SW41 Ti rotor, and the EV pellet was resuspended in 50 μl of 5% SDS in 50 mM Triethylammonium bicarbonate buffer (TEAB) buffer (Sigma). For immediate and functional assays EVs were stored in the fridge for up to a week to avoid any structural and functional changes to the vesicles, otherwise they were aliquoted and stored at –80°C.

### 2.3 Tunable resistive pulse sensing

Calibration particles (mode diameter 210 nm, 1 × 10^12^ particles/ml) and Nanopores (analysis range of 85–500 nm) were purchased from Izon Science Ltd (CPC200 and NP200 or NP150, respectively). All measurements were carried out using the qNano instrument (Izon Science Ltd, New Zealand) with a NP150 or NP200 nanopore, using the Control Suite software for data capture. 75 μl of PBS was added to the lower fluid cell via the side gaps. Care was taken to ensure that no air bubbles were introduced. The upper fluid cell was fixed in place and 40 μl of PBS was added. After every measurement, the upper fluid cell was washed out with the electrolyte buffer and a slight pressure was added to the system to remove any residual particles from the samples and prevent any cross‐contamination.

### 2.4 Transmission electron microscopy

#### 2.4.1 RPE and retinal organoids

RPE and retinal organoids were fixed in 2% glutaraldehyde in 0.1 M sodium cacodylate buffer pH 7.4 (TAAB Laboratories Equipment) at 4°C for a minimum of 24 h, then secondary fixed with 1% osmium tetroxide (Agar Scientific). Samples were dehydrated using graded acetone—25%, 50%, 75% (30 min each) and 100% acetone (2 × 60 min). Samples were impregnated with 25% resin in acetone, 50% resin in acetone, 75% resin in acetone (60 min each) and 100% resin for minimum of three changes over 24 h. Embedding was in 100% resin at 60°C for 24 h. Semi‐thin survey sections of 0.5 μm were cut and stained with 1% toluidine blue in 1% borax. Ultrathin sections (70 nm, approximately) were cut using a diamond knife on an RMC MT‐XL ultramicrotome or a Leica EM UC7 ultramicrotome. The sections were stretched with chloroform to eliminate compression and mounted on Pioloform‐filmed copper grids. Staining was performed using 2% aqueous uranyl acetate (approximately 30 min) and Lead Citrate (Leica UK) (7 min). The grids were examined on a Hitachi HT7800 transmission electron microscope using an Emsis Xarosa camera with Radius software.

#### 2.4.2 EVs and amyloid fibrils

Total 5 μl of EV and amyloid fibril suspensions in PBS were placed on a carbon‐coated and glow discharged copper grid (Gilder Grids) for 1–2 min. Subsequently the droplet was dried by touching to the edge of a hardened filter paper and negatively stained with 2% aqueous uranyl acetate (Agar Scientific) for a few seconds. Grids were examined on a Hitachi HT7800 transmission electron microscope using an Emsis Xarosa camera with Radius software.

### 2.5 TEM image analysis

TEM images (n = 10 per experimental group) of RPE subjected to the EV uptake study were analysed for various parameters of cell nuclei, including the area, eccentricity and perimeter, using Microscopy Image Browser (MIB) software (Belevich et al., 2016). For the retinal organoids treated with RPE EVs, mitochondria were analysed from 10 images per experimental group. Stereology was applied by sectioning the images to columns and mitochondria in each alternating column were segmented from the rightmost column; mitochondria that were positioned between two columns were segmented if they were placed on the left border of the columns selected for segmentation. Segmentation was conducted by outlining organelles to generate raw data. Raw data generated were the mitochondrial area, perimeter, and major (R) and minor axis (r) lengths. Mitochondria with areas below 1.0E‐03 μm^2^ were artifacts and not included. Form factor of mitochondria was used to assess the degree of branching and calculated using the formula: Form factor = Perimeter/4π × Area. Aspect ratio of mitochondria was used to assess the shape of the mitochondria and calculated using the formula: Aspect ratio = Major axis length (R)/Minor axis length (r). Data was analysed statistically for normality with Shapiro–Wilk test, followed by one‐way ANOVA and Dunnett's multiple comparisons test (parametric) or Kruskal–Wallis test and Dunn's multiple comparisons test (non‐parametric) using GraphPad Prism 9.0.0 software.

### 2.6 EVs fluorescent labelling, RPE EV in vitro uptake by RPE and retinal organoids and analysis by flow cytometry and fluorescence microscopy

RPE EVs were isolated from CCM using size exclusion chromatography, as described in Section 2.2, and concentrated to approximately 250 μl final volume by ultrafiltration (Amicon MWCO 10 kDa, Merck). An aliquot of the EV suspensions was lysed with SDS (1% final concentration), and protein concentration measured with the Pierce Micro BCA Protein Assay Kit (ThermoFisher) as per the manufacturer's instructions. ExoGlow‐Membrane EV Labelling Kit (SBI) was used to fluorescently label EV membranes. According to the manufacturer, the dye specifically labels intact EVs, while showing minimal background in an unbound state. The labelling reaction was carried out on an equivalent of 16 μg (confocal microscopy) or 25 μg (flow cytometry) of EV protein, according to the manufacturer's protocol. Unlabeled free dye was removed by precipitation with ExoQuick‐TC (SBI), as per the manufacturer's protocol.

For the flow cytometry analysis of EVs uptake by RPE cells, a visible EV pellet from ExoQuick‐TC precipitation was resuspended in Dulbecco's PBS (DPBS, Sigma) and the labeled EV suspension in cell culture media was added to fully confluent RPE cells grown on Matrigel coated 96‐well plate. Cells were incubated with the labeled EVs for 24 h in the standard cell culture conditions. Subsequently the cells were washed with PBS and dissociated with TrypLE Select Enzyme 10×. The enzyme was neutralised with PBS and cells were pelleted by centrifugation at 1000 × *g* for 3 min. Cells were resuspended in 1% exosome depleted FBS (ThermoFisher) in PBS and analysed by flow cytometry on an LSRFortessa (BD Biosciences), with 10,000 events collected for each sample. Untreated cells were used as a control. Data was analysed using FCS Express Software (BD Biosciences).

For confocal microscopy analysis of RPE EVs uptake by RPE cells and retinal organoids, the EV pellet was resuspended in 100 μl of DPBS and 20 μl aliquots were stored at –80°C until use. Three aliquots of labeled EVs were further purified by adding 1.2 ml of DPBS and ultracentrifugation at 130,000 × *g* for 1 h at 4°C in Optima Max‐XP Ultracentrifuge Beckman Coulter using the TLS‐55 S/N 17U4323 rotor. The EV pellets were resuspended in the initial volume of DPBS. Control reaction for the EV labelling and purification was carried out in parallel, using exactly the same procedure as for the EV samples, but DPBS was used instead of EV preparations. The presence of EVs after the labelling and purification procedure was validated by negative staining and transmission electron microscopy. Labelled EVs (2 aliquots of purified labeled EVs) and control reaction were added to the standard cell culture media to 1 well each of fully confluent RPE cells grown on Matrigel coated μ‐Slide 8 well‐high uncoated #1.5 polymer coverslips (ibidi). To investigate the internalised EVs only, fluorescently labelled EVs were incubated with cells for 5 h at normal culture conditions, after which the EV‐containing media was removed, and cells washed extensively with cell culture media. Time lapse images at approximately 1‐, 3‐, 5‐ and 24‐hours post EV‐exposure were acquired using an inverted Nikon A1R confocal microscope with NIS‐Elements software at controlled humidity and temperature of 37°C (excitation wavelength 488 nm, emission wavelength 595 nm). The images were taken at ×60 magnification with optical zoom at the exact same laser power settings with a 4× line average method. Images are shown at the exact same LUT brightness adjustment settings.

For fluorescence microscopy analysis of RPE EVs uptake by retinal organoids, one aliquot of purified labeled EVs was added to standard cell culture media to mature retinal organoids differentiation day 217, and these were incubated at standard cell culture conditions for 24 h. Organoids were washed in DPBS and live cell imaging was carried out using a Zeiss Lattice Lightsheet 7 fluorescence microscope (excitation wavelength 488 nm, emission wavelength 525 nm, Sinc3 100 μm × 1800 nm imaging beam). Post‐acquisition images were deconvolved and deskewed in Zen 3.5 software. A representative 3‐dimensional image is shown as a maximum intensity projection.

### 2.7 EV uptake study

#### 2.7.1 RPE EVs to RPE

EVs were isolated in aseptic conditions in PBS using size exclusion chromatography as described in Section 2.2. The assay design was to ensure that the levels of exogenous EVs per treatment were close to the RPE physiological levels of EVs (modelling co‐culture conditions and 1:1 ratio of donor:recipient cells). The volumes of CCM, cell numbers that the CCM was collected from (donor RPE) and the amounts of exogenous EVs per treatment were therefore determined considering the cell counts of RPE subjected to the treatment with exogenous EVs (host RPE). Cells were grown on either 0.3 or 1.1 cm^2^ Matrigel coated 0.4 μm PET hanging cell culture inserts (Merck) at a density of 450,000 cells/cm^2^. Exogenous EVs or an equivalent amount of PBS for controls were supplemented to the exosome depleted RPE cell media in 12 treatments over the course of 32 days in three biological replicates. A quarter of a transwell insert for each treatment category was collected and fixed immediately in 2% glutaraldehyde in 0.1 M sodium cacodylate buffer pH 7.4 for TEM ultrastructural analysis. The remaining cells were either fixed in 4% paraformaldehyde (Santa Cruz Biotechnology) for 60 min, washed in PBS and stored at 4°C until use or dissociated with TrypLE Select 10× (ThermoFisher) and collected for western blotting.

### 2.8 RPE EVs to retinal organoids

Retinal organoids at differentiation day 206 were treated with RPE EV suspensions in PBS or an equivalent amount of PBS for the control, by adding them to the standard cell culture media. EVs from approximately 3,500,000 RPE cells were harvested and purified as described in Section 2.2. Six treatments over the course of 2 weeks were performed. Bright field microscopy images of retinal organoids were taken at treatment day 0, 7 and 14. Some of the retinal organoids were fixed immediately after collection in 2% glutaraldehyde in 0.1 M sodium cacodylate buffer pH 7.4 for TEM ultrastructural analysis, while the rest were used for western blotting procedures or fixed in 4% paraformaldehyde for 30 min for immunofluorescence analysis.

### 2.9 Western blot

#### 2.9.1 RPE cells and retinal organoids

RPE pellets and retinal organoids were lysed with PhosphoSafe Extraction Reagent (Novagen) supplemented with Protease Inhibitor Cocktail (Roche). Lysates were prepared by pipetting and vortexing, followed by incubation on ice for 30 min. Samples were spun at 1000 × *g* for 10 min at 4°C and the supernatants collected and stored at –80°C until use. Protein concentration was determined using the Pierce BCA Protein Assay Kit (ThermoFisher) according to the manufacturer's instructions. 5–10 μg of total protein extract in samples containing the NuPAGE sample reducing agent and LDS sample buffer (Invitrogen) were heated at 70°C for 10 min and subjected to electrophoresis in NuPAGE 4% to 12% Bis‐Tris protein gels with MES sodium dodecyl sulphate (SDS) running buffer (Invitrogen). The gels were transferred onto polyvinylidene difluoride (PVDF) membranes using the iBlot 2 dry blotting system (Invitrogen) and the protein transfer visualised with a reversible Ponceau S staining (Sigma). Membranes were blocked with 5% dried skimmed milk in 1×Tris Buffered Saline (Santa Cruz Biotechnology)—0.1% Tween 20 (Bio‐Rad) (TBST) and incubated with primary antibodies (1 h at room temperature to overnight at 4°C) with gentle agitation (for primary and secondary antibody list see Table S1). Membranes were washed three times, 10 min each wash, and incubated with HRP conjugated secondary antibodies for 1 h at room temperature, following which the membranes were washed again three times at least 10 min each wash, and developed using SuperSignal West Pico PLUS Chemiluminescent Substrate (ThermoFisher). The chemiluminescent signal was detected with the Amersham Imager 600 (GE Healthcare) and the densitometry analysis was performed using ImageQuantTL (GE Healthcare). Data from 3–6 independent experiments was analysed for normality and statistical significance with the Schapiro Wilk test and one‐sample t‐test using GraphPad Prism 9.0.0. Mean ± SEM is shown on the representative graphs.

#### 2.9.2 EVs

**FIGURE 1:**
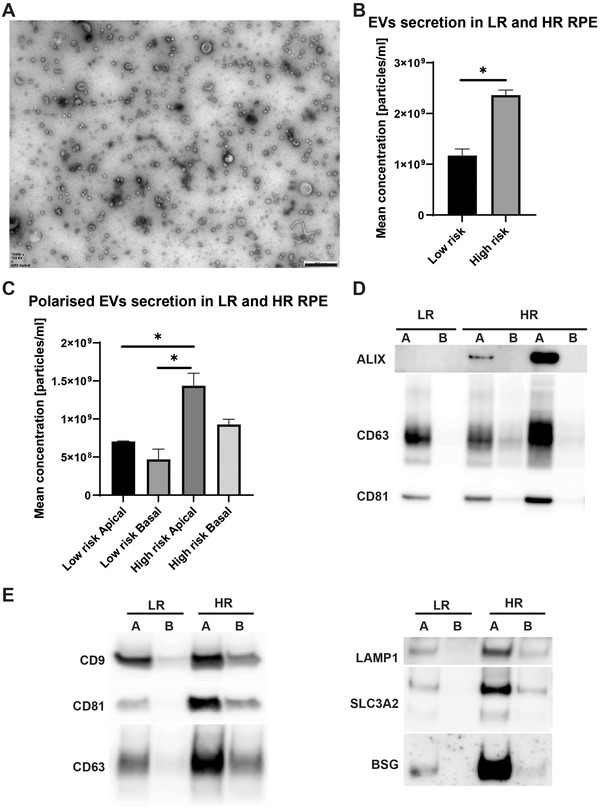
**Polarised EVs secretion in iPSC‐RPE**. (A) EVs were purified using size exclusion chromatography from CCM of human iPSC derived RPE grown on porous membrane supports that allow the cells to develop apical (A) and basal (B) polarity. A representative TEM image of apical EVs is shown. Scale bar 500 nm. (B) TRPS was used to quantify the EV samples, with four independent experiments carried out to characterise the rate of EVs secretion in high‐risk compared to low‐risk RPE. Cell DNA contents were used for normalisation. The same data sets were used to calculate average polarised and cumulative rate of secretion. Overall, high‐risk RPE secrete statistically significantly more EVs compared to the control low‐risk RPE. (C) High‐risk cells apical EV secretion is statically significantly enhanced compared to low‐risk apical and basal EV secretion. *p < 0.05. Mean with SEM shown. Statistical analysis by means of normality test, followed by unpaired *t* test or ordinary one‐way ANOVA with Tukey's multiple comparisons test. (D) EVs samples were immunoblotted for markers of exosomes (vesicles of endosomal origin) and ectosomes, with Alix, CD63 and CD81 being detected at various levels and indicating their particular enrichment in the apical fractions compared to the basal counterparts, a feature of RPE EVs described previously (Klingeborn et al., 2017). Note equal volumes of apical and basal EVs were loaded per sample to visualise the polarisation of EVs load and contents. (E) Equal EV protein amounts were screened by western blotting for the levels of exosomal (CD63 and LAMP1) and ectosomal (CD9, CD81, SLC3A2 and BSG) markers, as proposed previously (Mathieu et al., 2021).

**FIGURE 2:**
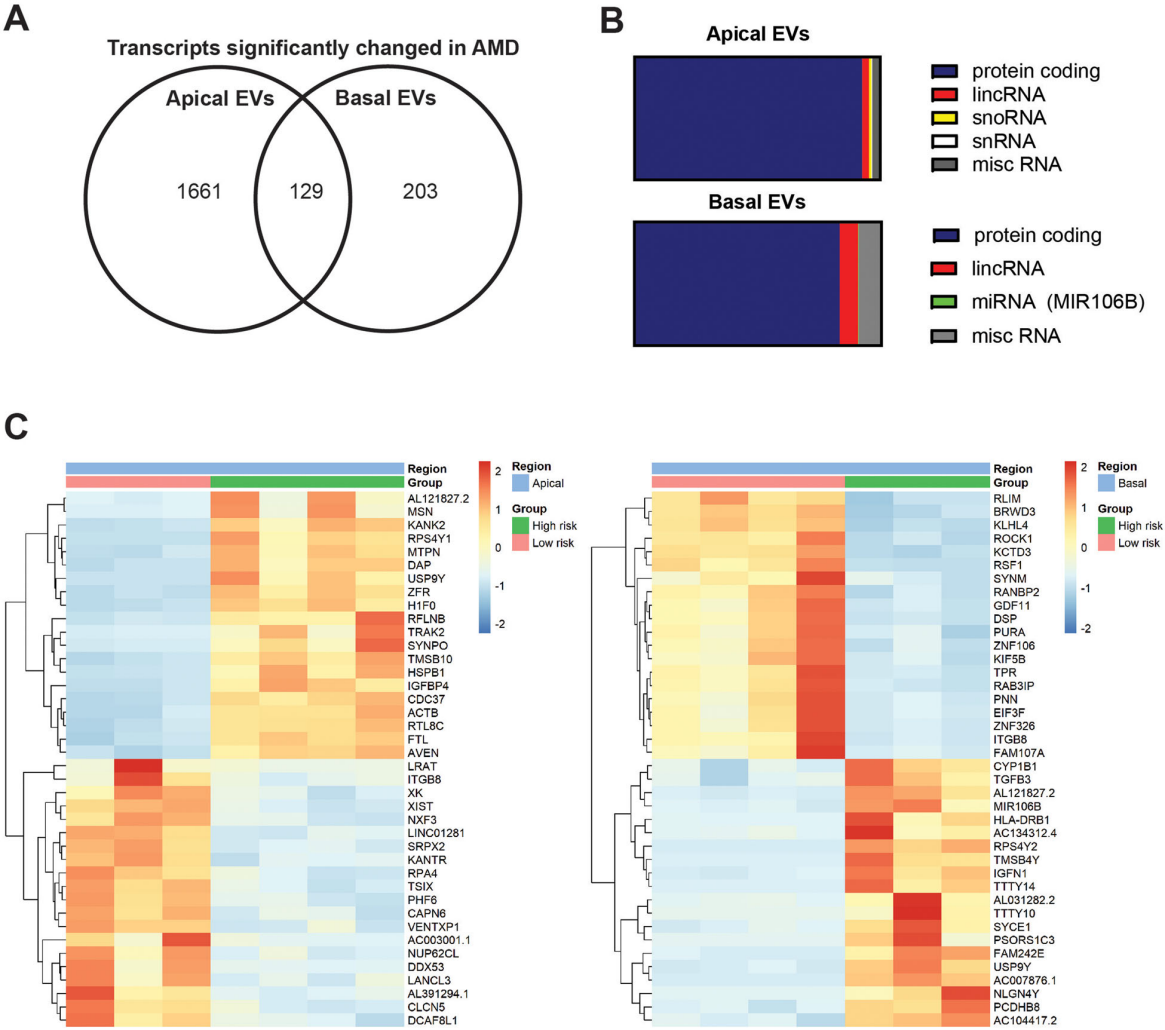
**Transcriptomics analysis of low and high‐risk RPE EVs**. (A) Numbers of transcripts significantly changed in high‐risk versus low‐risk RPE EVs (*p* value corrected < 0.05). (B) Distribution of transcript types among the differentially expressed transcripts. (C) Top 20 differentially expressed transcripts in apical/basal RPE EVs in high‐risk compared to low‐risk RPE EVs. Heatmaps show the normalised expression values from DESEQ2 at *p* value corrected < 0.05 cutoff.

Equal volumes of EVs for apical and basal EV fractions (amounts normalised by the donor cell DNA contents, Figure 1D) or 2 μg of EV protein (Figure 1E) as determined by Micro BCA (ThermoFisher) were subjected to sodium dodecyl sulphate‐polyacrylamide gel electrophoresis (SDS‐PAGE) using the Invitrogen NuPAGE system components without prior lysis. CD63, CD81, CD9, BSG, SLC3A2 and LAMP1 were detected in non‐reducing conditions, whereas samples for the detection of Alix were reduced with the NuPAGE sample reducing agent (Invitrogen) (for primary and secondary antibody details see Table S1). All samples were supplemented with the NuPAGE LDS reagent and heated for 10 min at 70°C. Electrophoresis, gel transfer, antibody incubation and chemiluminescent signal detection were performed as per the cell lysates western blot methodology, with the exceptions that extensive washes of 1 h in total were performed after the primary and secondary antibody incubations and the SuperSignal West Femto Maximum Sensitivity Substrate or SuperSignal West Pico PLUS Chemiluminescent Substrate (ThermoFisher) were used to develop the chemiluminescent signal.

#### 2.9.3 Immunofluorescence analysis

*RPE*

RPE cells grown on 24‐well PET hanging cell culture inserts (Merck) (pore size 0.4 μm) were fixed with 4% paraformaldehyde for 1 h at room temperature, washed in PBS and stored at 4°C in PBS until use. RPE membranes were incubated with blocking buffer containing PBS, 10% donkey serum and 0.3% Triton X‐100 (Sigma) for 1 h at room temperature, followed by incubation with primary antibodies (Vimentin, Abcam, ab92547) diluted in antibody dilution buffer (PBS, 1% donkey serum, and 0.1% Triton X‐100) overnight at 4°C. Following three washes with PBS, RPE cells were incubated with secondary antibodies (Donkey anti‐Rabbit Alexa 546, Life Technologies, A10040) diluted 1:800 in antibody dilution buffer for 1 h at room temperature. For nuclear staining RPE cells were incubated for 15 min with Hoechst (Life Technologies) diluted in PBS at 1:1000. RPE cells were mounted with Vectashield (Vector Laboratories) and sealed with a coverslip. Images were taken using the Axio Imager upright microscope with Apotome structured illumination fluorescence (Zeiss, Germany) and presented as a maximum intensity projection.

*Retinal organoids*

Organoids were fixed in 4% (w/v) paraformaldehyde for 30 min followed by several washes in PBS. After cryoprotection in 30% sucrose in PBS solution (Sigma) overnight, organoids were embedded in OCT (Cell Path Ltd.), sectioned (10 μm) on a cryostat (Leica Cm1860) and stored at –20°C until further use. For immunohistochemistry, retinal organoid sections were air‐dried, rinsed several times in PBS, and incubated in blocking solution (10% normal goat serum, 0.3% Triton X‐100 in PBS) for 1 h at room temperature. Primary and secondary antibodies were diluted in antibody diluent solution (1% bovine serum albumin (BSA), 0.3% Triton X‐100 in PBS). Primary antibodies (Table S2) were applied overnight at 4°C, followed by several washes in PBS, and an incubation with secondary goat antibodies conjugated either to Alexa488 or Cy3 (Jackson Immuno Research Laboratories) for 2 h at room temperature in the dark. After several washes in PBS, sections were mounted with VectaShield containing Hoechst. To detect non‐specific secondary antibody background staining, a control immunostaining was carried out by using the secondary antibody only. Images were acquired on a Zeiss Axio ImagerZ2 equipped with an Apotome.2 and Zen 2012 blue software (Zeiss, Germany). Single scan images were taken with a 10x/0.45 air objective and scanning of image stacks was performed with a 20x/0.8 air objective using a z‐axis increment of 0.49 μm. Images were adjusted for brightness and contrast in Adobe Photoshop CS6 (Adobe Systems) and representative images were shown as a maximum intensity projection.

*Immunofluorescence image quantification in retinal organoids*

Prior to immunofluorescence image quantitation non‐specific background staining (mainly in the centre of organoids) were removed using the MATLAB software (Mathworks). Immunofluorescence quantification was performed on 5–6 examples per condition as described previously (Dorgau et al., 2019) using MATLAB. After the final segmentation analyses, the percentage of either Pax6 or Vsx2‐positive cells were calculated and exported as Excel file for further analysis. Data were plotted and analysed statistically using GraphPad Prism, representing the mean and SEM for all conditions. Statistical significance was tested using one‐way ANOVA with Tukey's multiple comparisons test.

### 2.10 EVs RNA isolation

EV RNA was isolated using the Ambion *mir*Vana miRNA Isolation Kit (ThermoFisher) according to the manufacturer's protocol. EV suspensions were not treated with proteinase K and RNase, as these involve incubations at higher than ambient temperatures and small vesicles permeability increases rapidly from 36.5°C (Blicher et al., 2009). SUPERase‐In RNase Inhibitor (ThermoFisher) was added to the purified RNA samples at a final concentration of 1 u/μl. RNA was further precipitated with ethanol according to the following protocol: 0.1 volume of 3 M sodium acetate RNA grade, glycogen RNA grade (both ThermoScientific) to a final concentration of 1 μg/μl and three volumes of ice cold 100% ethanol molecular biology grade (Sigma) were added to the samples, thoroughly mixed and precipitated overnight at –80°C. Samples were centrifuged at 16,100 × *g*, 4°C for 30 min. The pellets were washed twice with 0.5 ml of ice cold 75% ethanol, spun at 4°C for 10 min each time. The traces of ethanol were removed by a brief centrifugation and the pellets air dried and re‐suspended in nuclease free water (Promega).

### 2.11 RNA sequencing

The EVs sequencing libraries were prepared using the SMART‐Seq Stranded Total RNA kit (Takara) following manufacturer's instructions choosing the ultra‐low input method with no sample fragmentation and sequenced on an Illumina NovaSeq 6000 at ∼40 million 2 × 100 bp reads per sample. The fastq files were trimmed using Trimmomatic (version 0.36), then aligned to the human reference genome using STAR version (2.5). The counts were then quantified using HTSEQ(0.8). A variance stabilizing transformations (VST) was applied to the data followed by PCA. The VST transformed data was further used to compute a distance. These diagnostic plots were used to identify outliers. Two samples were defined as outliers and were excluded from downstream analysis. The gene counts were imported into DESEQ2 (1.26) for differential expression analysis. The expression of differentially expressed transcripts is shown as heatmaps generated by Pheatmap (version 1.0.12).

### 2.12 Lipidomics

RPE cell and EV lipids were extracted in chloroform/methanol (2/1, v/v). Samples were vortexed and left on ice for 1 h following which the mixture was centrifuged at 2300 × *g* at 4°C for 10 min. The supernatant was removed, evaporated to dryness under a gentle stream of nitrogen gas and reconstituted in methanol containing 5 mM ammonium formate. Lipidomic analyses were performed using a Thermo Exactive Orbitrap mass spectrometer equipped with a heated electrospray ionization probe and coupled to a Thermo Accela 1250 UHPLC system. EV lipids were analysed in both positive and negative ion modes over the mass to charge (m/z) range 250–2000 at a resolution of 100,000. Lipids were separated on a Thermo Hypersil Gold C18 column (1.9 μm; 2.1 mm × 100 mm) maintained at 50°C. Mobile phase A consisted of water containing 10 mM ammonium formate and 0.1% (v/v) formic acid. Mobile phase B consisted of 90:10 isopropanol/acetonitrile (ACN) containing 10 mM ammonium formate and 0.1% (v/v) formic acid. The initial conditions for analysis were 65%A/35%B. The percentage of mobile phase B was increased from 35% to 65% over 4 min, followed by 65%–100% over 15 min, with a hold for 2 min before re‐equilibration to the starting conditions over 6 min. The flow rate was 400 μl/min. The raw LC‐MS data were processed with Progenesis QI software (version 2.1, Nonlinear Dynamics). Lipid identifications were made by searching against LIPID MAPS (www.lipidmaps.org/) and HMDB (http://www.hmdb.ca/) databases. Statistical analysis of differences in levels of lipids was performed by one‐way ANOVA and t‐test. Heatmaps were generated in Rstudio (R version 3.6.2, 2019‐12‐12) using package ‘pheatmap’ (https://www.R‐project.org/).

### 2.13 Proteomics

*RPE*

The protein containing pellet was air‐dried and reconstituted in SDS‐PAGE sample buffer. Proteins were separated by 1‐D SDS‐PAGE using a Mini Protean Tetra system (Bio‐Rad Laboratories). Samples were incubated at 95°C for 5 min in a reducing buffer (125 mM Tris‐HCl; 140 mM SDS; 20% v/v glycerol; 200 mM DTT and 30 mM bromophenol blue) prior to loading. Samples were electrophoresed at a constant potential of 200 V through a 15% w/v polyacrylamide resolving gel with a 4% w/v stacking gel. Gels were stained with Coomassie Blue (Bio‐Rad). Gel lanes were cut into 12 slices and each slice placed in distilled deionised water (50 μl). The water was then removed and the gel piece was treated with destain solution (10 μl of ACN/100 mM ammonium bicarbonate 1,1 v/v). The protein disulfide bonds were reduced by the addition of dithiothreitol (20 μl of 10 mM for 30 min) and alkylated by iodoacetamide (20 μl of 55 mM for 30 min incubation in the dark). Each gel slice was dehydrated in ACN. Trypsin (Roche) (0.2 μg/μl in 50 mM acetic acid) was added at a ratio of protein:trypsin 50:1 and the digestion allowed to proceed overnight at 37°C. The peptides were then extracted from the gel by addition of ACN and then dried under vacuum prior to resuspension in 50% methanol.

LC‐MS/MS analysis of peptides was performed in positive ion mode using a Thermo LTQ‐Orbitrap XL LC‐MSn mass spectrometer equipped with a nanospray source and interfaced to a Waters nanoAcquity ultra performance liquid chromatography (UPLC) system. The samples (5 μl) were initially desalted and concentrated on a 5 μm Waters Symmetry C18 180 μm × 20 mm trapping column. The peptides were then separated on a Waters BEH C18 nanocolumn (1.7 μm, 75 μm × 250 mm). Mobile phase A comprised 0.1% formic acid in water and mobile phase B comprised 0.1% formic acid in ACN. A gradient of 10%–40% ACN over 120 min was employed with a flow rate of 400 nl/min. Peptides were ionised at 3.5 kV source voltage. Acquisition was in data‐dependent (DDA) mode over the range m/z 300–2000 with the top 10 ions being fragmented using the lock mass setting for increased accuracy and comparability. Dynamic exclusion settings allowed a single repeat with a duration of 30 s, keeping a list of 500 ions. Charge state screening was enabled, rejecting unassigned and single positive charge states.

Protein identification was performed using Mascot (version 2.3, Matrix Sciences). The initial search parameters allowed for two trypsin missed cleavages, carbamidomethyl modification of cysteine residues, oxidation of methionine and acetylation of N‐terminal peptides and a false discovery rate (FDR) of 0.01. A mass tolerance of 20 ppm for the precursor ion first search and a tolerance of 6 ppm for main search were allowed along with a fragment mass tolerance of ±0.5 Da. A maximum 1% FDR was used for both protein and peptide identification. Protein identification was made from a minimum of two peptides per protein including at least one unique peptide. Identified contaminants were removed. Proteomic data were subjected to label‐free quantitative analysis with Progenesis QI for Proteomics software (version 4.2, Nonlinear Dynamics) to determine protein intensities. Analysis of the proteomic data was performed in Rstudio (packages ‘pheatmap’ and ‘factoextra’).

*EVs*

EV pellet suspensions were mixed 1:1 with 10% SDS solution (ThermoFisher). Protein digestion was carried out using S‐Trap (Protifi). Samples were heated at 95°C and sonicated to remove DNA/RNA. Samples were reduced with tris(2‐carboxyethyl)phosphine (5 mM final concentration, 15 min incubation at 55°C) and alkylated with Iodoacetamide (10 mM final concentration, 10 min incubation at room temperature). Each sample was then acidified with 12% phosphoric acid (final concentration of 2.5%, v/v), followed by addition of six volumes of loading buffer (90% methanol, 100 mM TEAB, pH 8) and loaded onto S‐Trap cartridges. The loaded cartridges were spun at 4000 × *g* for 30 s and washed three times with 90% loading buffer and the flow through being discarded. Retained proteins were digested with trypsin (Worthington), at a ratio of 10:1 protein to trypsin, in digestion buffer (50 mM TEAB, pH 8.5) for 3 h at 47°C. Peptides were released of the cartridge with three sequential washes; first with 50 μl of 50 mM TEAB, followed by 50 μl of 0.2% formic acid and finally with 50 μl of 50% ACN and 0.2% formic acid. The solution was frozen then dried in a centrifugal concentrator to a volume of ∼1 μl. The peptide sample was reconstituted in 8 μl 0.2% formic acid. A 5 μl of the peptide sample was loaded per LCMS run, peptides were separated with a 125‐min nonlinear gradient (3%–40% B, 0.1% formic acid (Line A) and 80% ACN, 0.1% formic acid (LineB)) using an UltiMate 3000 RSLCnano HPLC. Samples were first loaded/desalted onto a 300 μm × 5 mm C18 PepMap C18 trap cartridge in 0.1% formic acid at 10 μl/min for 3 min and then further separated on a 75 μm × 50 cm C18 column (Thermo EasySpray‐C18 2 μm) with integrated emitter at 250 nl/min. The eluent was directed to an Thermo Orbitrap Exploris 480 mass spectrometer through the EasySpray source at a temperature of 320°C, spray voltage 1900 V. The total LCMS run time was 150 min. Orbitrap full scan resolution was 60,000, RF lens 50%, Normalised ACG Target 300%. Precursors for MSMS were selected via a top 20 method. MIPS set to peptide, Intensity threshold 5.0 e3, charge state 2–7 and dynamic exclusion after one time for 35 s 10 ppm mass tolerance. ddMS2 scans were performed at 15,000 resolution, HCD collision energy 27%, first mass 110 m/z, ACG Target Standard. The data was acquired in DDA mode.

*EV mass spectrometry data analysis*

Protein identification and label‐free quantification was performed using MaxQuant Version 2.0.3.0. Triplicate injections were combined as one experiment. Trypsin/P set as enzyme; stable modification carbamidomethyl (C); variable modifications Oxidation (M), and Acetyl (Protein N‐term); Parent Mass Error of 5 ppm, fragment mass error of 10 ppm, maximum five modifications per peptide and two missed cleavages. Searches were conducted using the Uniprot human database (October 29, 2021, https://www.uniprot.org/uniprot/?query=proteome:UP000005640, concatenated to the Common Repository for Adventitious Proteins v.2012.01.01 (cRAP, ftp.thegpm.org/fasta/cRAP). Identifications were filtered at a 1% FDR at the peptide level, accepting a minimum peptide length of 7. Protein identification for total RPE‐derived EV protein list was based on proteins identified from a minimum of two peptides per protein including at least one unique peptide present in one sample (491 identified proteins). List of proteins was annotated by Gene Ontology Cellular Component (GOCC) using the Database for Annotation, Visualization and Integrated Discovery (DAVID) web‐based tool (https://david.ncifcrf.gov/). For comparison between apical and basal EVs, proteins identified in at least two different sample types were included (418 proteins included). Protein quantification was performed using razor and unique peptides with at least one peptide identified by MS/MS. ‘Match between runs’ was enabled. LFQ intensities were extracted for each protein/sample preparation (based on injections n = 3) and protein quantified if present in more than one sample.

### 2.14 Ingenuity pathway analysis (IPA)

Transcriptomic and proteomic data were analysed for canonical pathway enrichment using IPA (Qiagen) Core Analysis based on the expression log ratio. Corrected for multiple testing *p* value cutoff was set at 0.05. Graphical presentation of data was performed using GraphPad Prism 9.0.0 software.

### 2.15 Inflammation assays

#### 2.15.1 THP‐1 cell culture conditions

Human monocytic THP‐1 cells (ATCC #TIB‐202) were cultured in RPMI‐1640 containing 10% fetal bovine serum (Gibco, Thermo Scientific), 0.05 mM β‐mercaptoethanol (Gibco, Thermo Scientific), and 1% penicillin‐streptomycin (Gibco, Thermo Scientific). Cell cultures were maintained at 37°C with 5% CO_2_ and split 1:4 at a seeding density of 106 cells. For differentiation, THP‐1 cells were seeded in 6‐well plates and treated with 2.5 ng/ml phorbol‐12‐myristate‐13‐acetate (PMA) for 48 h. Prior to stimulation, cells were allowed to rest in culture media for 24 h. THP‐1‐differentiated macrophages were stimulated with the same volumes of RPE EVs, to model the physiological EV concentrations produced by the high and low risk lines (average particle count for low‐risk apical 1.2E+07, low‐risk basal 1.3E+07, high‐risk apical 2.0E+07 and high‐risk basal 1.3E+07), for 18 h before harvesting.

#### 2.15.2 Quantitative real‐time PCR analysis

Treated THP‐1 cells RNA was isolated using RNeasy kit (Qiagen). Total RNA (500–1000 ng) was used to synthesize cDNA with the QuantiTect Reverse Transcription Kit 15 (Qiagen). Quantitative real‐time PCR analysis was performed using the Applied Biosystem SYBR Green Mastermix and analysed by Applied Biosystems 7500 Real‐Time PCR systems. Results for gene expression of inflammatory cytokines: Tumor Necrosis Factor alpha (*TNF alpha*) and chemokine (C‐C motif) ligand 2 (*CCL2*), and inducible nitric oxide synthase (*iNOS2*) were normalised to the gene expression of TATA‐binding protein 1 (*TBP1*) and expressed as fold change relative to gene expression of control basal EVs treated cells using the comparative CT method (ΔΔCT). *TNF alpha* primers were from QuantiTect primer assays (Qiagen). *CCL2, iNOS2* and *TBP1* primer sequences were as shown in Table S3 and they were purchased from Invitrogen.

### 2.16 Angiogenesis assays

#### 2.16.1 3D‐tubulogenesis assay

Endothelial colony forming cells (ECFCs) cultured as a cell monolayer were treated overnight with apical and basal low‐risk and high‐risk RPE EVs. EVs were derived from cell conditioned media from 200,000 RPE cells (per replicate) to model 1:1 ratio of exposure RPE:ECFCs. Additionally, to assess the functional effect of EVs, dose‐response experiments were carried out at different ratios of RPE EVs – ECFCs (i.e., 1:4 and 1:2). Exosome depleted media was used to harvest EVs secreted by RPE cells over 72 h and EVs were purified using size exclusion chromatography as described in Section 2.2. After treatment, ECFCs were detached and counted. The angiogenesis μ‐Slide (Ibidi) was used to assess 3D tube formation capacity. An ECFC suspension was mixed in a 2:3 ratio with Matrigel (Corning) and dispensed into slide to form a 10 μl Matrigel droplet containing 15,000 cells (final density of 1.5 million ECFCs per ml). Angiogenesis μ‐Slides were left in the incubator for 30 min to enable Matrigel polymerisation, and then covered with 50 μl of EGM‐2 media (Lonza). Tube‐like structures were assessed after 48 h, using the EVOS Cell Imaging system (Thermo Fisher). Analysis of the tube area was performed using ImageJ.

#### 2.16.2 3D‐bead sprouting assay

ECFCs were treated overnight with EVs isolated from low and high‐risk RPE cells as described above. EVs were harvested from 500,000 RPE cells (per replicate) to model 1:1 ratio of exposure RPE:ECFCs. ECFCs were counted, fluorescently labeled with SP‐DiOC18(3) (ThermoFisher) and used to coat the microcarrier Cytodex beads (Amersham Pharmacia Biotech, Piscataway, NJ) in a flow cytometry tube (pluriSelect Life Science, Leipzig Germany) containing 1.5 ml of EGM2 for 4 h at 37°C and 5% CO_2_. The tube was mixed gently every 30 min to maximise bead coating. After 4 h, the ECFC‐coated beads were resuspended in 5 ml EGM‐2 media containing EVs, the contents were transferred to a T25 flask and incubated overnight 37°C and 5% CO_2_. The following day, beads were embedded in Fibrin gel solution in a clear bottom 96‐well plate (Thermo Fisher Nunc MicroWell) containing 2 mg/ml Fibrinogen, 0.15 U/ml Aprotinin and 0.625 U/ml Thrombin (Merck). The fibrin clot was allowed to form by incubating the plate for 5 min at room temperature and 15 min at 37°C. Finally, beads were supplemented with 150 μl EGM2 containing 3300 Human Lung Fibroblasts (passage 6) per well of a 96‐well plate, dispensed dropwise and incubated for 5 days at 37°C and 5% CO2. Image acquisition for single beads was carried out using Leica TIRF microscope (Leica microsystems) and sprouting efficiency was assessed by counting the number of sprouts using LasX platform (Leica Microsystems).

### 2.17 Protein aggregation assay

Real time quaking‐induced conversion (RT‐QuIC) assay was performed as described previously (Groveman et al., 2018; Kurzawa‐Akanbi et al., 2021) with modifications. Black 96‐well plates with a clear bottom (Thermo Fisher Scientific) were preloaded with six silica beads (1 mm, Thistle Scientific) per well. Seeded α‐synuclein aggregation was conducted by loading 15 μl of 0.2 μm filtered PBS (negative control), 15 μl of α‐synuclein fibrils (positive control; 5 mg/ml – 1:20,000 dilution), and EVs samples (1.5E+07 particles for all samples as determined by TRPS) to the wells containing 85 μl of the reaction mix to give final concentration of 40 mM phosphate buffer (pH 8.0), 175 mM sodium chloride, 0.1 mg/ml recombinant full‐length α‐synuclein (Proteos) and 10 μM thioflavin T (ThT). Recombinant human α‐synuclein was filtered through a 100 kDa MWCO filter immediately prior to use (Amicon, MERCK). The plate was sealed with a Nunc sealing tape (Thermo Fisher Scientific) and incubated at 37°C in a BMG FLUOstar Omega plate reader with cycles of 1 min shaking (400 rpm double orbital) and 1 min rest throughout the indicated incubation time. ThT fluorescence measurements (448 nm excitation and 482 nm emission; gain 1000, bottom read) were taken every 30 min.

## 3 RESULTS

### 3.1 High‐risk AMD RPE are characterised by enhanced polarised secretion of EVs

Our previous work has shown that high‐risk AMD RPE cells are characterised by pronounced defects in autophagy, manifested by the expansion of the endolysosomal system, lysosomal membrane instability and reduced proteolytic activities, alongside with the deposition of key lysosomal components, such as Cathepsin D in drusen‐like deposits (Cerniauskas et al., 2020). We hypothesised that the lysosomal deficiency may affect vesicular trafficking, and in particular the EVs release in these cells, as the multivesicular bodies can either deliver their intraluminal vesicles to the lysosomes for degradation or to the cell surface giving rise to exosomes (Kalluri & LeBleu, 2020). The block in autophagy in the high‐risk RPE cells that we identified might potentially lead to the re‐direction of cargo through promoting secretion.

We therefore investigated the rate of EVs release by the high‐risk RPE cells in comparison to low‐risk RPE in a cell culture system, described previously (Johnson et al., 2011), that allows the cells to develop their natural polarisation essential for their normal functions in photoreceptor maintenance (apical surface) and contact with the choroidal vasculature across Bruch's membrane (basal surface). CCM was collected from apical and basal chambers of low and high‐risk RPE cells and EVs were isolated using size exclusion chromatography. Transmission electron microscopy confirmed the enrichment in small EVs (Figure 1A). Tunable resistive pulse sensing (TRPS) was used to determine the EV concentration and size. Our results showed that the high‐risk RPE lines secreted on average twice as many EVs to the apical and basal side compared to the control low‐risk lines (Figure 1B); results that were validated across 4 independent experiments. The overall increased EV release in high‐risk cells was mostly due to the increased secretion from the apical side (Figure 1C). TRPS also validated the enrichment in small EVs in the RPE‐derived EV samples (average ± standard deviation of modal size from eight independent samples measurements per group): 102.2 ± 13.47 nm (low‐risk apical), 101.9 ± 19.64 nm (low‐risk basal), 112.3 ± 4.399 nm (high‐risk apical) and 100.5 ± 7.801 nm (high‐risk basal). Furthermore, western blot analysis for the endosomal system derived EV markers (exosomes), such as CD63 and ALIX, and ectosomal marker CD81 (Kowal et al., 2016; Mathieu et al., 2021), revealed their comparatively higher abundance in the apical side released EVs than the basal side counterparts (Figure 1D). This feature has been observed previously (Klingeborn et al., 2017) and is suggestive of the high‐level polarisation of RPE cellular functions, including differential proteomes of secreted vesicles. Western blot analysis of equal EV protein amounts for markers of exosomes (CD63 and LAMP1) and ectosomes (CD9, CD81, SLC3A2 and BSG) (Mathieu et al., 2021) demonstrated their higher levels in the high‐risk apical and basal EVs compared to the low‐risk apical and basal EVs (Figure 1E), implying that secretion of both, exosomes and ectosomes is enhanced in the high‐risk RPE cells compared to low‐risk RPE cells.

Overall, our results indicate that the high‐risk AMD RPE cell EV secretion is significantly enhanced compared to the control low‐risk RPE cells.

### 3.2 Altered composition of high‐risk AMD RPE cell EVs

#### 3.2.1 EVs transcriptomics indicates enrichment of genes involved in oxidative stress, angiogenesis, and cytoskeletal signalling in AMD‐RPE cells

EVs carry a portfolio of biological material, including proteins, nucleic acids and lipids (Kalluri & LeBleu, 2020). We therefore sought to characterise the molecular components of EVs isolated from CCM of low and high‐risk RPE cells, apical and basal fractions, to gain more insight into the potential disease signalling via the RPE EV secretome. Transcriptomic analysis was carried out on RNA isolated from the EV enriched samples. Over 50,000 transcripts were detected in both apical and basal EV samples. Statistical analysis of high‐risk versus low‐risk outputs at the significance level set at p value corrected <0.05, revealed 129 differentially expressed transcripts that were common to the apical and basal EVs, and with 1661 specific to the apical EVs and 203 present in the basal EVs only (Figure 2A and Table S4). The vast majority of the differentially expressed transcripts were protein coding, with a small proportion of non‐coding RNAs (Figure 2B). The top 20 changed transcripts in apical and basal high‐risk versus low‐risk comparisons are shown in Figure 2C. Interestingly, multiple transcripts altered in either apical or basal high‐risk RPE EVs encoded proteins involved in cytoskeletal functioning (*MSN, KANK2, MTPN, SYNPO, TMSB10, ACTB, TMSB4Y*), microtubule stabilization (*CAPN6*), regulation of perinuclear actin network and nuclear shape through interaction with filamins (*RFLNB*), formation of intermediate filaments (*SYNM*) and desmosomes (*DSP, PNN*).The apical high‐risk RPE EVs were also enriched in transcripts encoding molecular chaperones (*HSPB1, CDC37*). Interestingly, miRNA MIR106B, linked to the control of vascular endothelial growth factor (VEGF) expression (El Baroudi et al., 2011), was upregulated in the basal high‐risk EVs (Figure 2C).

**FIGURE 3:**
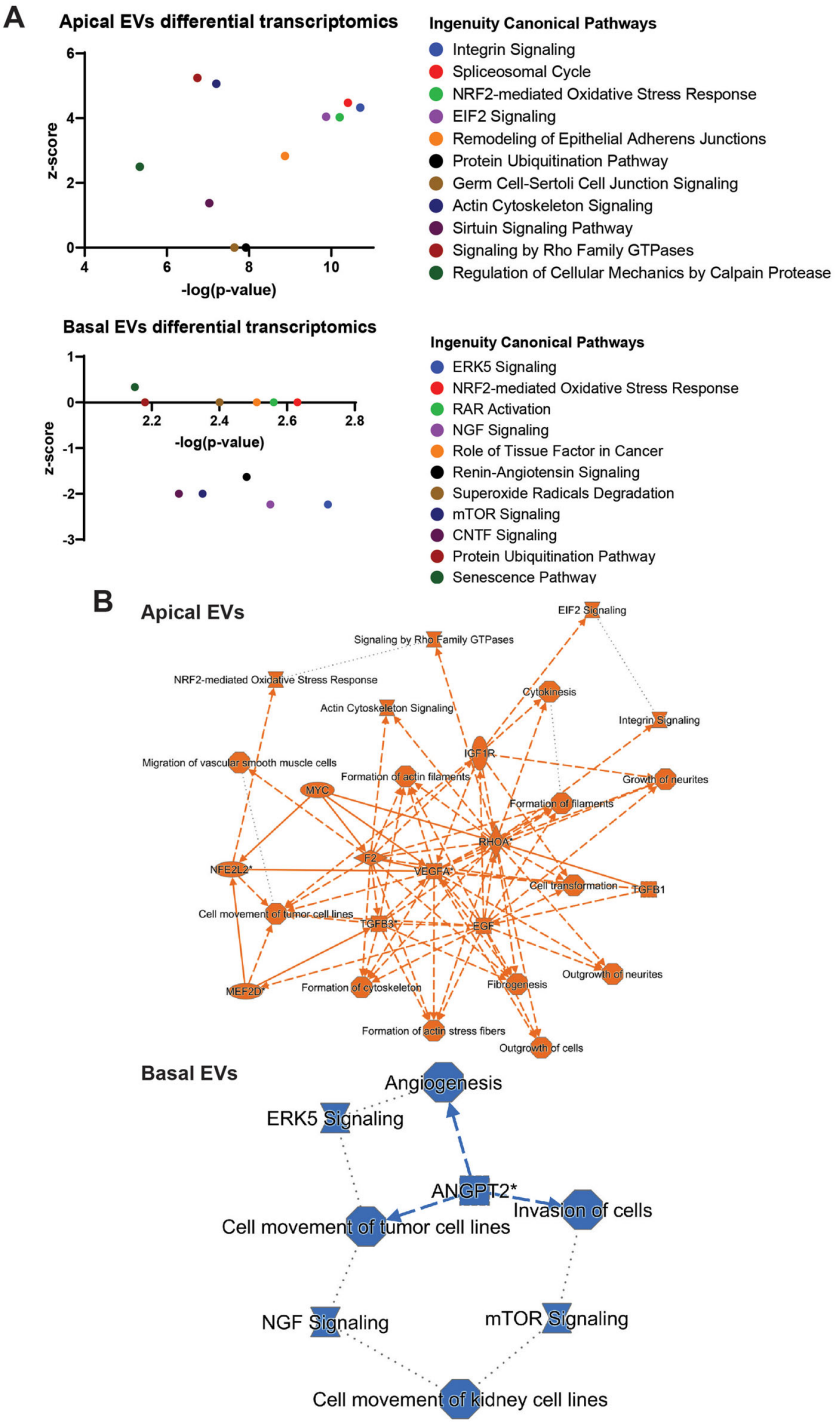
**IPA analysis of differentially expressed transcripts in high‐risk versus low‐risk RPE EVs**. Ingenuity IPA analysis of differential transcriptomics results indicate that EVs may be involved in disease related mechanisms in high‐risk AMD RPE, including oxidative stress response, angiogenesis, actin cytoskeleton signaling, protein homeostasis and senescence. (A) Statistically significantly enriched pathways against the inferred activation z‐score. (B) IPA graphical summaries representing networks of the major pathways identified as the most significant in the differential transcriptomics data for the apical and basal high‐risk compared to low‐risk RPE EVs. The edges show direct (solid lines) and indirect (dashed lines) interactions between molecules in the network. Inferred edges are shown with dotted lines. Orange colour—predicted activation, blue colour—predicted inhibition state. Interactions are inferred from using the Ingenuity knowledge base.

IPA analysis was carried out on the differentially expressed transcripts (*p* value corrected < 0.05) for the apical and basal high‐risk versus low‐risk EVs to identify networks of significantly overrepresented biological pathways. In the apical EVs integrin signalling, NRF2 mediated oxidative stress response, EIF2 signalling, actin cytoskeleton signalling and regulation of cellular mechanics by calpain protease were among the significantly changed pathways, whereas in the basal EVs oxidative stress response, major signalling pathways that control cell survival/apoptosis and cell metabolism (ERK5, NGF, CNTF, mTOR), signalling pathways related to angiogenesis (RAR and renin‐angiotensin) and senescence constituted the most changed biological processes (Figure 3A).

Overall, as underpinned by the IPA graphical summaries shown in Figure 3B, signalling through the most important pro‐angiogenic factor, VEGFA, was central to the mechanistic network identified for the apical high‐risk EVs, coupled with NRF2‐mediated oxidative stress response and actin‐cytoskeleton signalling. As inferred by IPA, these pathways were enhanced in the apical EVs transcriptomics. On the other hand, angiogenesis regulated by angiopoietin growth factor 2 (ANGPT2), predicted to be decreased, was at the core of the signalling conveyed by the high‐risk basal EVs.

#### 3.2.2 EVs proteomics indicates enrichment of cell stress, dysregulation of extracellular matrix, angiogenesis and drusen formation proteins in AMD‐RPE cells

**FIGURE 4:**
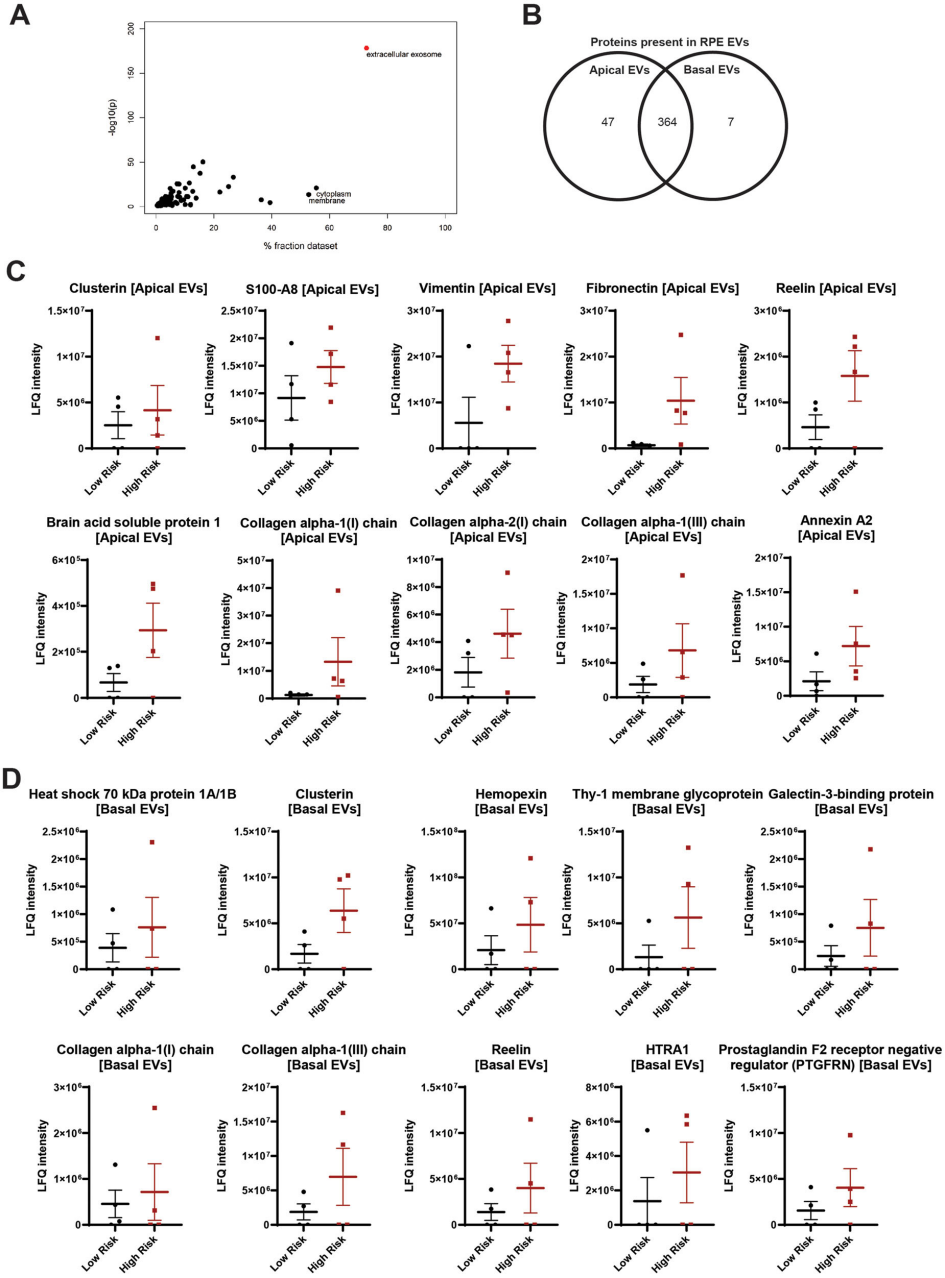
**Proteomics analysis of low and high‐risk RPE EVs**. (A) Enrichment of exosomal proteins in the RPE‐derived EV samples. All proteins identified with >1 unique peptide were included. Proteins were annotated by Gene Ontology Cellular Component (GOCC) and the term enrichment using the DAVID web‐based tool (https://david.ncifcrf.gov/). Results are presented as a correlation of dataset fraction annotated by the given term and Benjamini–Hochberg corrected *p*‐value of the enrichment test. In red, extracellular exosome GOCC dataset with >70% dataset fraction detected. (B) Venn diagram showing counts of proteins identified in apical and basal RPE EVs. For details see Table S5. (C) Label free quantitation (LFQ) intensities of selected proteins present in apical and (D) basal RPE EVs. Mean with SEM shown.

The global proteomic analysis of EVs contents resulted in identification of 491 proteins (based on identification from a minimum of two peptides per protein including at least one unique peptide) in low and high‐risk apical and basal EVs cumulatively. As annotated by the Gene Ontology (GO) Cellular Component, the identified proteome consisted of >70% exosome specific proteins, indicating high purity of the EV isolations (Figure 4A). Comparison of apical and basal EVs contents, based on 418 proteins detected in at least two different sample types, revealed 364 proteins common for both EV fractions, 47 specific for apical EVs and seven present in the basal EVs only (Figure 4B and Table S5). GO terms analysis of all 418 detected proteins revealed overrepresentation of pathways involved in extracellular signalling, and a significant enrichment of processes linked to AMD RPE pathogenesis, such as oxidative stress, complement activation, innate immune response, stress response, amyloidosis, unfolded protein binding and angiogenesis (Table S6). A total of 111 proteins were quantified in the apical EVs and 71 proteins in the basal EVs (Table S7), with subsets being specifically enriched in high‐risk compared to low‐risk RPE EVs (Table S8). Among those, high‐risk apical EVs showed a trend towards increased levels of stress‐related proteins such as clusterin, peptidyl‐prolyl cis‐trans isomerase A (PPIA), Thy‐1 membrane glycoprotein, heat shock 70 kDa protein 1A/1B and S100‐A8, also suggestive of pro‐inflammatory signalling. Furthermore, cytoskeletal and extracellular matrix (ECM) proteins including vimentin, protein kinase c and casein kinase substrate in neurons 1 (PACSIN 1), fibronectin, and reelin (extracellular matrix protease), were enriched in high‐risk RPE EVs. Various collagen chains, for example collagen alpha‐1(I), alpha‐2(I) and alpha‐1(III) chains were also specifically enriched in both apical and basal high‐risk RPE EVs. Importantly, given the neovascular AMD phenotype of the high‐risk RPE donors used in this study, angiogenesis linked brain acid soluble protein 1, EGF‐like repeat and discoidin I‐like domain‐containing protein 3 (EDIL 3) and aquaporin‐1 were also more abundant in the high‐risk RPE EVs (Figure 4C and Table S8). Correspondingly, the basal high‐risk EVs also showed enrichment of cell stress associated proteins (clusterin, hemopexin, heat shock 70 kDa protein 1A and 1B, Thy‐1 membrane glycoprotein and galectin‐3‐binding protein), ECM components such as collagen, ECM proteases—reelin and serin protease HTRA1, and prostaglandin F2 receptor negative regulator (PTGFRN) with a role in angiogenesis (Figure 4D). Importantly many of these enriched proteins are known components of sub‐RPE deposits/drusen (e.g., clusterin, vimentin, collagen, S100‐A8 and fibronectin), and are associated with RPE dysfunction and stress, therefore constituting a risk for propagating the AMD phenotype to adjacent RPE or photoreceptor cells (Crabb, 2014; Crabb et al., 2002; Sakaguchi et al., 2002). Furthermore, the enrichment of HTRA1 protease in the basal EVs, validated its biological relevance to AMD risk, beyond its genetic link to AMD development, and supported its likely contribution to extracellular debris accumulation and neovascularisation via its proteolytic cleavage activity (Fritsche et al., 2016). Clusterin and fibronectin are known cleavage targets of HTRA1, hence their abundance in the high‐risk EVs may constitute favourable environment for extracellular debris formation (Lin et al., 2018). As other proteins specifically enriched in the apical or basal high risk RPE EVs (SLC2A1, RELN, TPI1, EEF2, COL1A2, SQSTM1 and LGALS3BP) have been associated in the literature with AMD, this further validates our findings (Hongisto et al., 2017; Kim et al., 2014; Kim et al., 2018; Rinsky et al., 2021; Viiri et al., 2013; Xu et al., 2019; Zuo et al., 2012).

Overall, our proteomics analysis revealed the specific enrichment of proteins linked to cell stress, angiogenesis, cytoskeletal and ECM signalling, and drusen formation in high‐risk RPE EVs.

#### 3.2.3 EVs lipidomics indicates ganglioside shedding in AMD‐RPE cells

**FIGURE 5:**
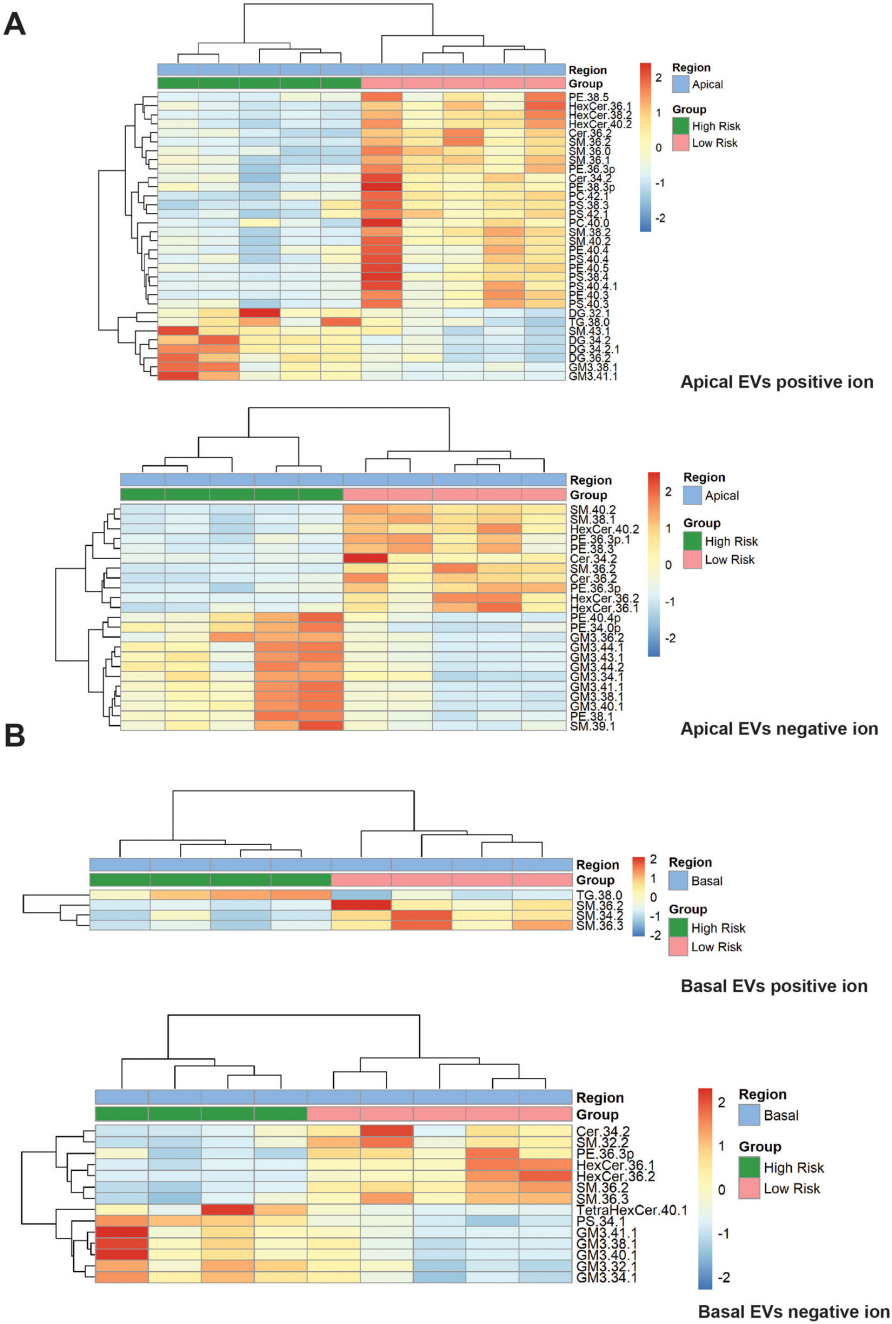
**Lipidomics analysis of low and high‐risk RPE apical and basal EVs**. Heatmaps show supervised clustering of statistically significantly changed lipids from positive and negative ion mode analyses, based on *t*‐test and *p* value < 0.05 cutoff.

Lipidomics analysis was carried out on the apical and basal high‐risk and low‐risk RPE EVs and revealed changes in various sphingolipid and glycerophospholipid classes in the high‐risk RPE EVs (Figure 5). Interestingly, we observed a significant enrichment in several GM3 ganglioside species in both apical and basal EVs. GM3 is the major ganglioside in the RPE (Daniotti et al., 1994). Gangliosides are particularly abundant in nervous tissue and play important roles in neurodevelopment (Yu et al., 2012). The lack of GM3 synthase activity leads to the loss of GM3 and its biosynthetic derivatives, and manifests as autosomal recessive infantile‐onset symptomatic epilepsy syndrome associated with developmental stagnation and blindness (Simpson et al., 2004). The vision loss is thought to be specifically associated with central nervous system and optic nerve involvement (Farukhi et al., 2006). There is evidence showing that GM3 ganglioside accelerates protein aggregation and in particular, neurodegeneration linked amyloid beta and alpha‐synuclein aggregation, with the latter demonstrated to be induced by exosomal lipids (Grey et al., 2015; Yamamoto et al., 2005). As amyloid beta assemblies are a common component of drusen (Anderson et al., 2004), it is likely that the RPE EV contained GM3 gangliosides are involved in AMD drusen build up process.

Increased abundance of neutral lipids (di‐ and triacylglycerols) was also observed in both apical and basal high‐risk RPE EVs (Figure 5). As these lipids are commonly present in lipoprotein particles that have similar biophysical properties to the EVs, including the size and density (Menard et al., 2018), it cannot be excluded that these particles were co‐purified with EVs. Lipoproteins and EVs may, however, actively interact; for example, it has been shown that the uptake of EVs may be changed when lipoproteins are present in the environment (Menard et al., 2018). Overall, the higher abundance of neutral lipids in the high‐risk EV enriched samples may indicate their association with a differential AMD RPE cell signalosome. Given the age‐related accumulation of neutral lipids in the Bruch's membrane and the vast neutral lipid contents of drusen, the current results might be indicative of the ‘oil spill’ processes (Curcio et al., 2011).

### 3.3 RPE EV contents reflect changes in the cells of origin

**FIGURE 6:**
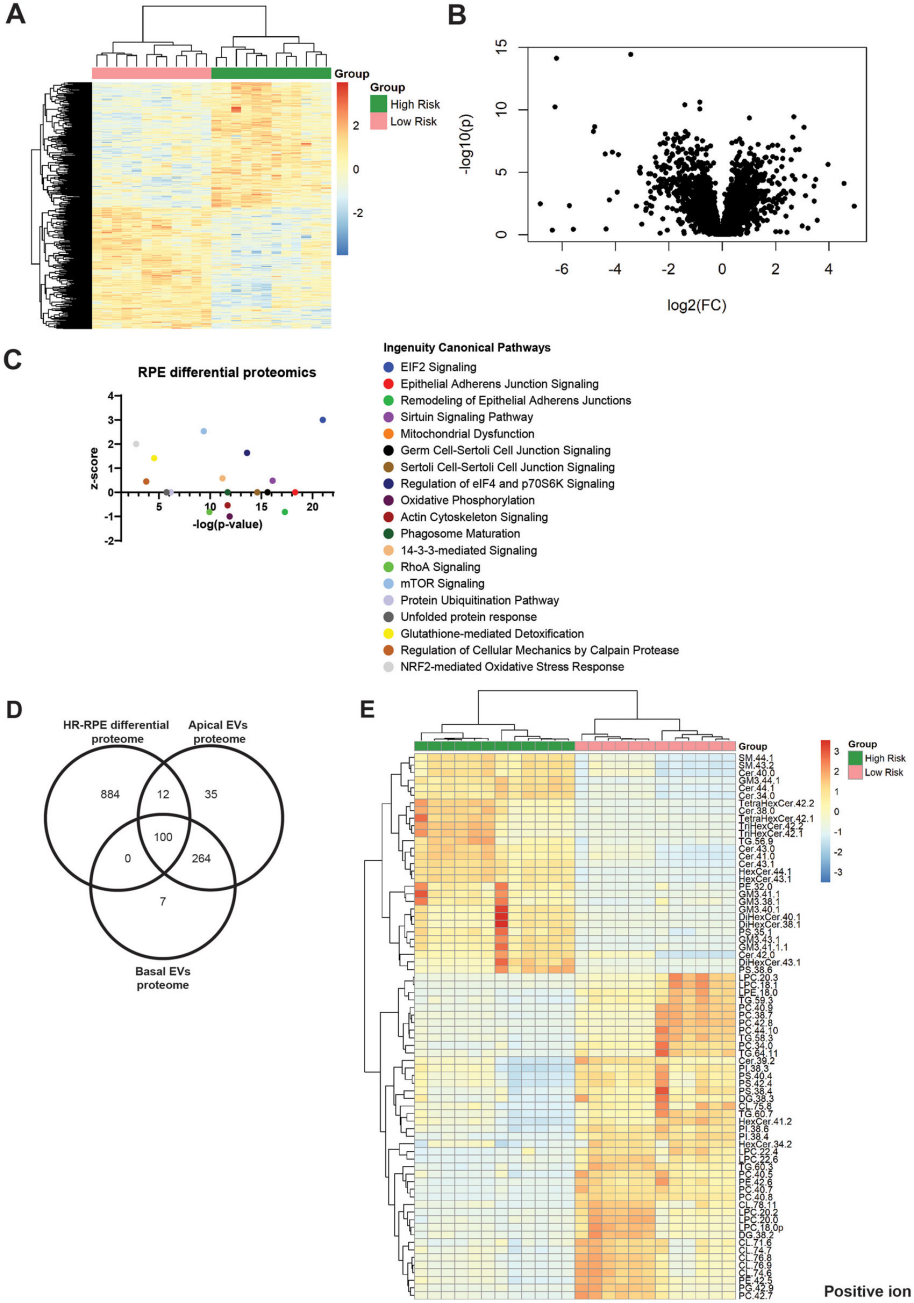
**Proteomics and lipidomics of low and high‐risk RPE cell lines**. (A) Heatmap represents supervised clustering of proteins in high‐risk versus low‐risk RPE significantly altered at *p* value corrected <0.05. (B) Volcano plot representing protein expression changes between high‐risk and low‐risk RPE. (C) Ingenuity IPA analysis of low and high‐risk RPE differential proteomics indicates statistically significantly enriched canonical pathways (‐log *p* value > 1.3) against an inferred activation z‐score, that is, increased or decreased biological function. (D) Venn diagram showing counts of proteins identified in apical and basal RPE EVs, and their overlap with the differentially expressed proteins in high‐risk versus low‐risk RPE at *p* value corrected < 0.05. (E) Lipidomics analysis of low and high‐risk RPE lines. Heatmap shows supervised clustering of statistically significantly changed lipids from positive ion mode mass spectrometry analysis, based on *t*‐test and *p* value < 0.0001 cutoff.

We next assessed the proteomes and lipidomes of the low and high‐risk RPE cells lines to test whether the EVs contents reflect metabolic changes in the cells of origin. Proteomics analysis of the RPE cells identified differentially expressed proteins in high‐risk versus low‐risk RPE (*p* value corrected < 0.05, Figure 6A,B). The subsequent IPA analysis suggested the canonical pathways that were enriched in the differential proteomes, with EIF2 signalling, mitochondrial dysfunction, actin cytoskeleton signalling, RhoA signalling, mTOR signalling, unfolded protein response, glutathione‐mediated detoxification, regulation of cellular mechanics by calpain protease and NRF2‐mediated oxidative stress response constituting the major significantly changed pathways in the high‐risk compared to low‐risk RPE (Figure 6C). This analysis revealed a high degree of overlap with the EVs proteomics results, in particular for the oxidative and protein stress response, cytoskeletal organisation, angiogenesis and senescence, indicating that many molecules involved in the disease process are present in the cell secretome in the extracellular milieu and are available to interact with the adjacent cells (Figure 6D and Table S9).

Differential RPE cell lipidomics revealed statistically significant decreases in neutral glycerolipids (di‐ and triacylglycerols) as well as multiple glycerophospholipid species including mitochondrial cardiolipin in the high‐risk AMD RPE compared to low‐risk control RPE (Figure 6E and Figure S1). There were also statistically significant increases in molecular species of sphingolipids such as ceramides, hexosylceramides and GM3 gangliosides. Interestingly, the enrichment in GM3 ganglioside was clearly in common with the EVs lipidomics findings (Figure 5) and suggestive of the ganglioside shedding to the extracellular milieu.

Overall, the comparison of results from the multi‐omics analyses of high and low‐risk RPE EVs and the RPE cells of origin revealed that the disease related molecular pathways are shared between the cells and the EVs.

### 3.4 High‐risk AMD RPE EVs confer disease phenotype to control RPE cells

**FIGURE 7:**
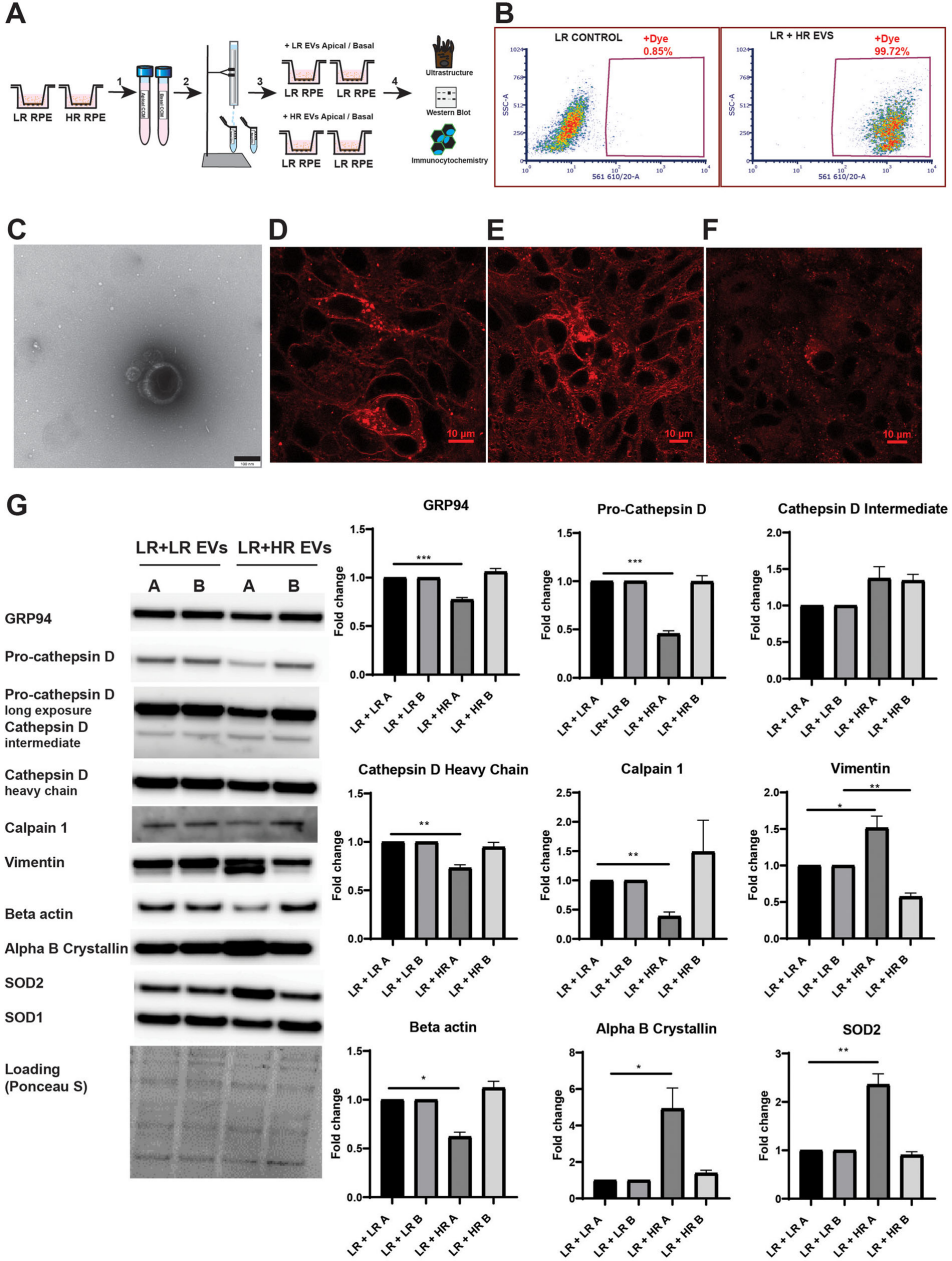
**RPE EV transfer assays**. (A) EVs were purified from low‐risk and high‐risk apical and basal CCM using size exclusion chromatography (1, 2). Low‐risk RPE cells were then exposed to the low/high‐risk apical/basal EVs in 12 treatments over the course of 32 days (3). The functional impact of exogenous EVs on the recipient cells was analysed using TEM, for the cell ultrastructure, western blot and immunocytochemistry for the expression of specific protein markers (4). (B) EVs uptake efficiency was assessed by EV membrane fluorescent labelling, RPE exposure to the labelled EVs for 24 h and flow cytometry. (C) Fluorescently stained EVs visualised on transmission electron microscopy. Scale bar 100 nm. Fluorescently stained EVs were exposed to RPE cells and confocal microscopy images were taken at (D) 1 h and (E) 3 h post‐exposure. (F) To investigate the internalised EVs only, cells were washed after 5 h post EV exposure and confocal microscopy images were taken 24 h post‐exposure. Scale bar 10 μm. (G) Western blot analysis indicates that markers of ER stress (GRP94), lysosomal proteolytic function (Cathepsin D) and stress responses (SOD2, alpha B crystallin) are changed in the low‐risk RPE treated with the high‐risk apical EVs. Cytoskeletal changes are also pronounced in these cells as indicated by the reduced levels of beta actin and a proteolytic cleavage of vimentin. Experiment was performed in triplicates. Mean + SEM shown; *p < 0.05, **p < 0.01, ***p < 0.001. A,apical; B, basal; LR, low risk; HR, high risk.

As the analysis of EVs contents indicated that cytoskeletal maintenance, formation of actin stress fibers, oxidative stress response and angiogenesis may be mediated by the EV signalling in the high‐risk AMD RPE, we explored whether these features could be transferred to control RPE cells. To evaluate this biologically functional potential of EVs, we isolated EVs from the apical and basal CCM of high and low‐risk RPE and exposed low‐risk control RPE to the exogenous EVs in two types of experiments (Figure 7A‐F). To validate the ability of RPE to take up EVs, we performed in vitro EVs uptake assays, whereby purified low and high‐risk EVs were stained with a fluorescent membrane dye, visualised by TEM (Figure 7C), and incubated with low‐risk control RPE overnight. Flow cytometry was used to detect and quantify the RPE‐EV uptake events, as the fluorescently labeled EV membranes are distributed to the cellular membranes through internalization. Our data indicated that fluorescently labeled EVs were successfully delivered to the recipient RPE cells after a 24‐h incubation (Figure 7B). In agreement with these observations, confocal microscopy of RPE cells exposed to fluorescently labeled EVs showed clear cell membrane staining at 1 and 3 h post EV exposure (Figure 7D‐E). To investigate the internalised EVs only, the cells were washed after 5 h post EV exposure and imaged using confocal microscopy 24 h after exposure. Cytoplasmic and perinuclear fluorescent staining was clearly visible, suggestive of the exogenous EVs incorporation to the endolysosomal system (Figure 7F and Figure S2).

We then exposed low‐risk control RPE to the exogenous low and high‐risk apical and basal EVs in 12 treatments over the course of 32 days, with the assay design modelling 1:1 ratio of donor to recipient cells. Screening for specific protein markers by western blot revealed that control RPE exposed to the apical high‐risk EVs were characterised by a significant reduction of the ER resident chaperone GRP94, half of the control amounts of pro‐cathepsin D precursor and a 30% decrease in levels of the mature heavy chain of cathepsin D, one of the major lysosomal proteases (Figure 7G). This was coupled by an average 5‐fold increase in the expression of alpha B crystallin, a small heat shock protein exhibiting potent anti‐apoptotic functions by attenuating oxidative stress and ER stress induced cell death (Kannan et al., 2016). Mitochondrial manganese dependent superoxide dismutase 2 (SOD2), but not superoxide dismutase 1 (SOD1), was also significantly elevated in cells treated with high‐risk apical vesicles, indicating induced oxidative stress response in those cells, notably of mitochondrial origin.

Remarkably, we observed that cytoskeletal beta actin protein expression was decreased by approximately 40% and vimentin was proteolytically cleaved (Figure 7G), both features of a dramatic disruption of the cytoskeleton of the cells. As vimentin is a substrate for proteolytic cleavage by caspases during apoptosis (Byun et al., 2001), we immunoblotted the RPE cell lysates for caspase 3 and poly (ADP‐ribose) polymerase (PARP) cleavage fragments, serving as markers of apoptosis (Oliver et al., 1998). Although, no cleavage fragments of caspase 3 and PARP were detected, we observed a significant reduction of pro‐caspase 3 (approximately 40%) in the RPE exposed to the apical high‐risk EVs compared to the RPE exposed to the apical low‐risk EVs, and a trend towards increased levels of PARP in cells exposed to both apical and basal high‐risk EVs (Figure S3), further indicating genotoxic stress and activation of cell survival mechanisms.

The loss of intermediate filaments due to calpain‐mediated vimentin cleavage has been shown to drive pyroptotic cell death, which is linked to inflammasome activation (Davis et al., 2019). We, therefore, looked for the evidence of calpain activation in the RPE lysates exposed to exogenous EVs, which is manifested by calpain degradation (autolysis) (Azuma et al., 1997). Indeed, the levels of calpain were approximately 60% decreased in the lysates of RPE exposed to high‐risk apical EVs (Figure 7G), indicating calcium induced calpain activation and vimentin cleavage.

**FIGURE 8:**
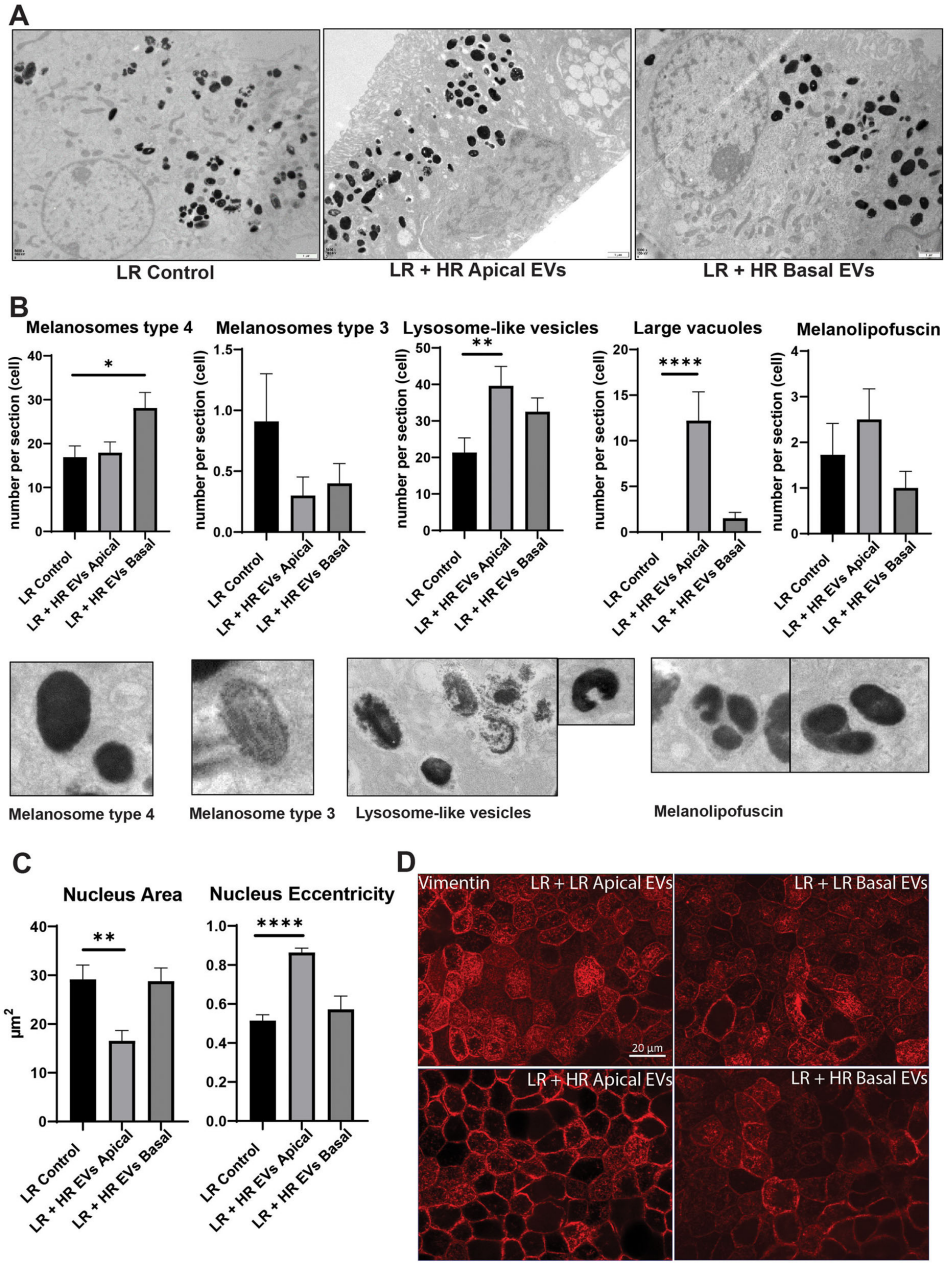
**High‐risk RPE EVs confer disease phenotype to control RPE cells**. (A) RPE ultrastructural features on TEM post‐EV exposure. LR Control—low risk PBS treated. (B) Melanosomes, vesicles with visible melanin degradation (melanolysosomes or lysosome‐like vesicles), large vacuoles and melanolipofuscin granules were quantified with Mean + SEM presented. Data was analysed for normality, followed by ANOVA and Dunn's multiple comparisons test. *p < 0.05, **p < 0.01, ****p < 0.0001. (C) Parameters of the cell nuclei were analysed with MIB Helsinki software, followed by statistical analysis (normality test followed by ANOVA with Dunnett's or Dunn's multiple comparisons test where appropriate). Mean + SEM shown, **p < 0.01, ****p < 0.0001. (D) Immunofluorescence analysis of vimentin expression in low‐risk RPE treated with low/high risk apical/basal EVs. Note the pronounced loss of intermediate filaments in high‐risk apical EVstreated RPE cells.

Treatment with basal high‐risk RPE EVs did not show any major alterations in the protein markers tested, but vimentin was significantly reduced in the treated cells compared to cells treated with basal low‐risk RPE EVs. Consistent with these findings, TEM analysis showed dramatic changes in the cell morphology of RPE exposed to the high‐risk apical EVs, with no major changes to the cells treated with the high‐risk basal EVs, with the exception of significantly increased number of mature melanosomes (Figure 8). Low‐risk cells exposed to the high‐risk apical EVs developed numerous stress vacuoles and showed a high degree of melanin degradation as indicated by the presence of vesicles with various stages of melanin disintegration (melano‐lysosomes or lysosome‐like vesicles) (Gouras et al., 2011); features in common with our earlier ultrastructural observations in high‐risk AMD RPE (Cerniauskas et al., 2020; Hallam et al., 2017) (Figure 8A,B). Furthermore, the high‐risk AMD RPE EVs induced pronounced changes to the cell nuclei, with the average nucleus area in those cells being 50% lower compared to the control, and the eccentricity, which is a measure of how ‘round’ an ellipse is, was significantly higher than for the controls and close to the value of 1, indicating an abnormally ‘squashed’ morphology of the nucleus (Figure 8A,C).

Given the pronounced changes in the cytoplasm visible on TEM images of the cells exposed to the high‐risk apical EVs, generally severely compromised cellular organisation, hardly any visible mitochondria and vesicular structures, we hypothesised a loss of cellular integrity due to cytoskeletal disruption. Consistent with these observations, immunofluorescence analysis of vimentin showed pronounced loss of intermediate filaments in high‐risk apical EVs treated cells in comparison to cells treated with either low risk EVs or basal high‐risk EVs (Figure 8D).

Together these results demonstrate that high‐risk AMD RPE EVs are biologically functional and are potent inducers of disease phenotype, with cytoskeletal disruption, changes in nuclear morphology and lysosome‐like vesicles number, protein and oxidative stress constituting the major pathological events following the exposure to the apical side‐released high‐risk AMD RPE EVs. No major pathological changes were detected in high‐risk basal RPE EVs treated control RPE cells, indicating differential contents of this pool of EVs and underscoring the high‐level polarisation of RPE cells.

### 3.5 High‐risk AMD RPE EVs induce pathological changes in the retinal organoids containing photoreceptor cells

As in the in vivo retina, the RPE apical side faces the photoreceptor cells, we sought to determine the effect of apical high‐risk AMD EVs on mature retinal organoids, exhibiting fully developed inner and outer segments of the photoreceptor cells. We first evaluated the ability of retinal organoids to uptake RPE EVs, and we incubated fluorescently labelled RPE EVs with retinal organoids for 24 h in standard cell culture conditions. Fluorescence microscopy analysis showed fluorescent staining of the global structure of the organoid, including the presumptive photoreceptor cell layer (Figure S4). Fluorescent signal from membrane and cytoplasmic locations was visible (Figure S4B) throughout the organoids, indicative of the exogenous fluorescently labelled EVs cellular uptake.

**FIGURE 9:**
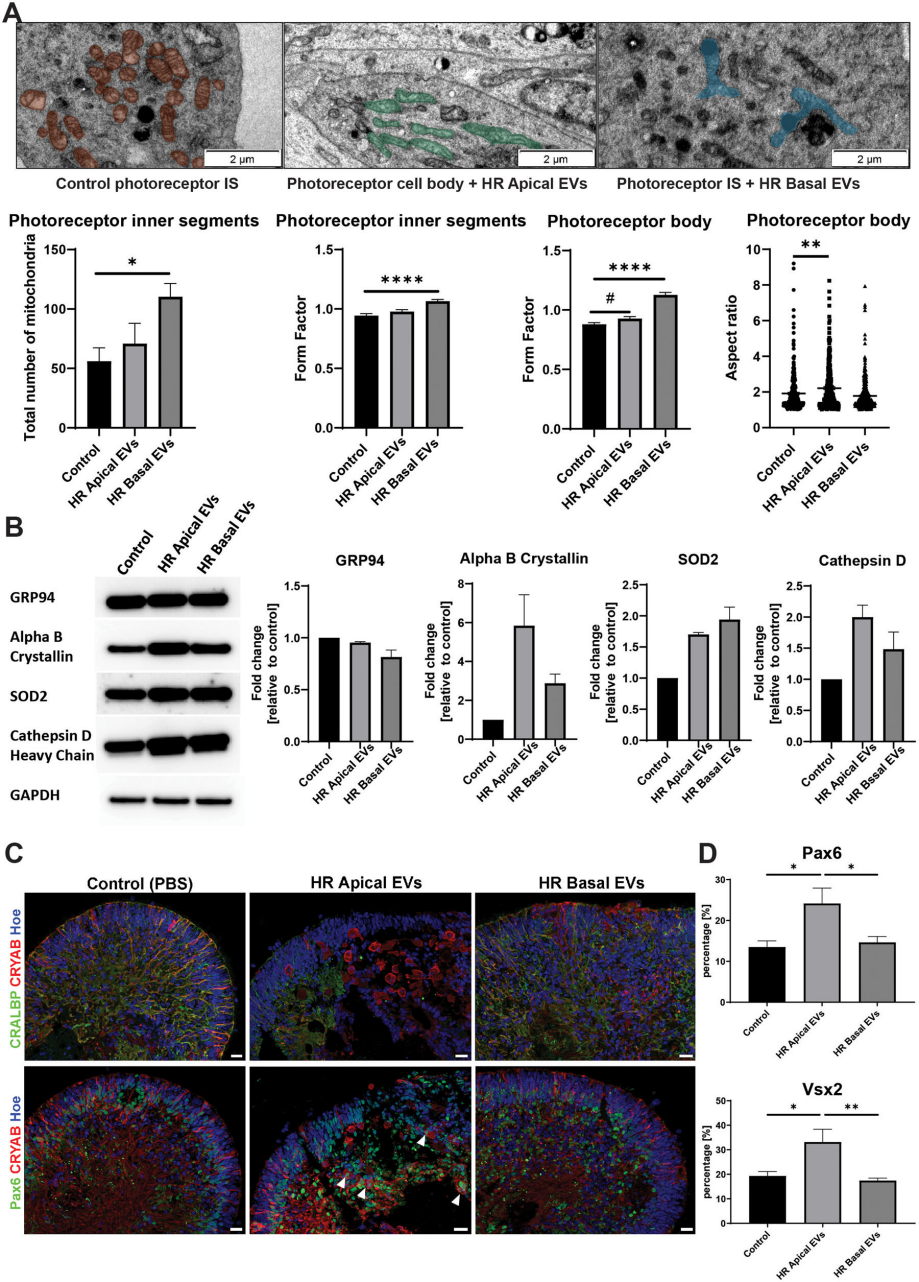
**High‐risk RPE EVs confer stress signals to the photoreceptors**. (A) Photoreceptor cell bodies and inner segments ultrastructural analysis by TEM shows changes in mitochondrial shape and organization (shaded structures) following the exposure to apical and basal high‐risk RPE EVs. MIB analysis of TEM images revealed statistically significant changes in mitochondria count, form factor (measure of mitochondria shape complexity and branching) and aspect ratio (measure of the length to width ratio). Statistical analysis by ANOVA followed multiple comparisons testing. Mean with SEM presented, 10 images per experimental group analysed, *p < 0.05, **p < 0.01, ****p < 0.0001, ^#^ *p* = 0.0506. Scale bar 2 μm. IS, inner segments. (B) Western blot analysis demonstrates increased expression of protective alpha B crystallin, SOD2 and cathepsin D, with no changes in ER stress marker GRP94. Mean with SEM of fold changes respective to GAPDH expression and relative to the control, n = 5. (C) ICC analysis of alpha B crystallin (CRYAB), Müller glia CRALBP marker, and neural progenitor cells Pax6 marker protein expression in retinal organoids treated with apical or basal high‐risk EVs, in comparison to PBS vehicle treated control. Cell nuclei were counterstained with Hoechst. Scale bar 20 μm. Arrowheads denote enlarged cells immunopositive for alpha B crystallin and Pax6 protein expression. (D) Quantification of Pax6 and Vsx2 positive staining. Mean + SEM shown, n = 8, *p < 0.05, **p < 0.01.

Retinal organoids (differentiation day 206) were then treated with apical or basal high‐risk RPE EVs alongside a PBS vehicle treated control in six doses, as part of the routine cell media changes. Post‐exposure TEM analysis revealed structural abnormalities in the mitochondria in both apical and basal high‐risk RPE EVs treated photoreceptor cells. Apical high‐risk EVs treatment associated with significantly higher degree of mitochondria elongation and branching in the photoreceptor cell bodies, reflected by the increased aspect ratio and form factor measurements, respectively (Figure 9A). The treatment with basal high‐risk EVs led to an increase of mitochondria number in the photoreceptor inner segments and significant changes in the mitochondrial shape complexity and branching in both photoreceptor bodies and inner segments (Figure 9A). Changes in mitochondrial shape, that are attributed to mitochondrial dynamics and fission/fusion events, reflect the metabolic status of the cell. Clustering of elongated mitochondria has been observed in aged monkey RPE, and metabolic and oxidative stress were proposed to underlie these changes (Gouras et al., 2016). Mitochondrial elongation appears to be a compensatory mechanism and confers resistance to apoptotic cell death (Jahani‐Asl et al., 2007).

As changes in mitochondrial shape may be early indicators of metabolic and oxidative stress, we screened the retinal organoid homogenates for cell stress markers by western blot. Notably, cytoprotective alpha B crystallin showed approximately 6‐fold change in levels in apical high‐risk RPE EVs treated retinal organoids compared to the control and approximately 3‐fold increase in levels in basal high‐risk EVs treated organoids (Figure 9B). Oxidative stress responsive SOD2 also showed a trend towards increased levels in both apical and basal EVs treated retinal organoids, as well as the major lysosomal protease cathepsin D (Figure 9B). The endoplasmic reticulum resident chaperone GRP94 was however not changed. Together with the changes in mitochondrial dynamics, these cellular alterations are suggestive of oxidative stress insult, as well as the burdened lysosomal degradative capacity.

To gain insight into any potential abnormalities to the laminated structure of the retinal organoids, we performed immunofluorescence analysis for retinal cell markers, such as recoverin (photoreceptors), gamma‐synuclein (retinal ganglion cells), CRALBP (Müller cells), GFAP (astrocytes), as well as alpha B crystallin, validated retinal cell stress marker. Irregular abnormal morphology, with disrupted neuroepithelium and hollow areas were characteristic for retinal organoids treated with high‐risk apical EVs; features absent in high‐risk basal EVs treated and vehicle treated control organoids, presenting normal morphology with continuous neuroepithelium (Figure S5). Staining for alpha B crystallin in high‐risk apical treated EVs revealed the presence of unusual enlarged, balloon‐like cells, primarily in areas affected by morphological abnormalities (Figure 9C and Figure S5B). Those cells and gross areas of their presence were mostly negative for Müller glia and astrocytes markers immunostaining, with only occasional cells co‐stained for CRALBP and alpha B crystallin (Figure 9C, Figure S5B,C). Müller glia have been observed to transiently enter the neurogenesis pathway in response to injury, via formation of Pax6‐positive intermediate progenitor cells and migration to different retinal laminae giving rise to different retinal cell types (Ahmad et al., 2011). Immunostaining for Pax6 and Vsx2, proliferative retinal progenitor cell markers, revealed the presence of cells positive for alpha B crystallin and Pax6 or Vsx2 (Figure 9C,D and Figure S5D,E), indicative of the activation of the regenerative pathway through alpha B crystallin neuroprotective glial cell response, as shown previously in other types of neurodegeneration (Hampton et al., 2020). Taken together, our data show that high‐risk apical EVs induce cell stress in retinal organoids, with evidence of activation of cytoprotective regenerative mechanisms via Müller glia cell response.

### 3.6 High‐risk AMD RPE EVs enhance angiogenesis

To investigate whether the altered transcriptomic and proteomic profiles of high‐risk AMD RPE EVs, suggestive of angiogenic signalling, had a biologically functional role in new vessel formation, we studied the effect of RPE EVs on endothelial colony forming cells (ECFCs) tube formation and vessel sprouting. Sprouting vessels are a result of endothelial cells responding to angiogenic stimuli and constitute a fundamental process in blood vessel formation (Potente et al., 2011). ECFCs were treated with RPE EVs for 18 h at concentrations modelling exposure at 1:1 ratio (cell counts ratio) of RPE to ECFCs. Stimulation of ECFCs with high‐risk apical RPE EVs resulted in statistically significantly enhanced tubulogenesis when compared to the assay control (PBS) and low risk apical and basal RPE EVs (Figure S6A). In agreement with these observations, high‐risk apical RPE EVs significantly increased the number of angiogenic sprouts when compared to the assay control and low risk apical and basal RPE EVs (Figure S6B). This pro‐angiogenic effect of high‐risk apical EVs appeared to be mediated by their contents and was further enhanced by their increased amounts, as shown by dose‐response experiments (Figure S7). Interestingly, exposure of ECFCs to the basal high‐risk RPE EVs did not enhance tubulogenesis nor significantly stimulated vessel sprouting, compared to the low‐risk RPE EVs stimulation. These findings suggest that apical high‐risk RPE EVs constitute angiogenic stimuli for new vessel sprouting and these results are in strong agreement with the EVs differential transcriptomic profiling. Our observations imply an important role of RPE EVs in the process of neovascularization in AMD by driving abnormal blood vessel growth from the choroid into the subretinal space.

### 3.7 High‐risk AMD RPE EVs stimulate protein aggregation

The protein and lipid contents of high‐risk RPE EVs suggested their potential contribution towards AMD drusen and subretinal drusenoid deposits (pseudodrusen) accumulation. In particular, the specific enrichment of GM3 ganglioside species in high risk apical and basal RPE EVs could have a role in amyloid β‐protein (Aβ) fibrils assembly, as demonstrated previously (Matsuzaki et al., 2010; Yamamoto et al., 2005). Aβ fibrils and oligomeric assemblies are very common components of drusen and considered to form a scaffold onto which other components including ApoE, complement proteins and CFH could bind to form drusen (Anderson et al., 2004; Isas et al., 2010). Aβ assembly is known to be highly neurotoxic, triggering neuroinflammation, angiogenesis, cytotoxicity and cell death, and growing evidence suggests its involvement in retinal pathology in AMD (Lynn et al., 2017). GM3 gangliosides also strongly induce aggregation of the α‐synuclein protein, which analogously to the Aβ protein, assembles into β‐sheet enriched amyloid fibrils and is known to accumulate in neurodegeneration and in the aging brain and retina (Gaspar et al., 2018; Grey et al., 2015; Leger et al., 2011). In order to evaluate the protein aggregation inducing properties of the GM3‐rich high‐risk RPE EVs, we employed a kinetic assay wherein α‐synuclein fibril formation is monitored by fluorescence intensity of ThT, which specifically binds to the amyloid β‐sheet assemblies. The same EV particle counts from low‐risk and high‐risk apical and basal RPE EVs preparations were incubated with normal recombinant α‐synuclein, alongside positive (preformed α‐synuclein fibrils) and negative (PBS) controls. The ThT fluorescence markedly increased in the presence of high‐risk basal RPE EVs, at a level comparable to fibril generation with α‐synuclein pre‐formed fibrils, and to a lesser extent in the presence of apical high‐risk RPE EVs (Figure S8A). TEM analysis revealed the generation of α‐synuclein fibrils and on many occasions intact or disrupted EVs trapped in the fibril net, indicative of their contribution to fibril formation (Figure S8B). Importantly abundant amyloid fibrils of similar morphology were found in sub‐RPE deposits of human specimen (Isas et al., 2010). No significant ThT fluorescent signal from α‐synuclein fibrils was detected in the presence of low‐risk apical and basal RPE EVs. These results indicate that basal high‐risk RPE EVs, and to a lower degree apical high‐risk RPE EVs, can induce protein amyloid β‐sheet formation, which may contribute to the characteristic amyloid assembly formation and biogenesis of drusen.

### 3.8 High‐risk apical AMD RPE EVs are immunostimulatory

Local and systemic inflammation is a recognised feature of AMD (Kauppinen et al., 2016). Given the identified cell stress related contents of high‐risk RPE EVs, we hypothesised that these EVs constitute pro‐inflammatory signals and may activate peripheral myeloid cells. In support of this hypothesis, macrophage invasion in regressing drusen accompanied by degenerative changes in the RPE has been previously observed (Sarks et al., 1999). To investigate this immunomodulatory potential of RPE EVs, THP‐1‐derived macrophages were stimulated with apical or basal high and low‐risk EVs for 18 h and the induction of *TNF alpha*, *CCL2* and *NOS2* inflammatory associated genes was analysed. This analysis indicated that high‐risk apical EVs induce the expression of pro‐inflammatory cytokine *TNF alpha*. In addition, the apical high‐risk RPE EVs induced a higher expression of all pro‐inflammatory genes analysed, when compared to the basal counterpart (Figure S9). These results indicate that the apical high‐risk RPE EVs may be involved in the recruitment of immune cells to the subretinal space, contributing to the neovascularization and photoreceptor atrophy (Tan et al., 2020).

## 4 DISCUSSION

Our data provide strong evidence that patient specific AMD RPE are characterised by enhanced polarised secretion of EVs. Multi‐omics analyses of the EVs contents revealed that the cargo of AMD RPE EVs differed from control RPE EVs and reflected the molecular changes in the RPE cells of origin. Our bioinformatic analyses of the differential EVs contents suggested that AMD RPE EVs may mediate disease features such as the oxidative stress response, angiogenesis, cytoskeletal dysfunction and extracellular debris accumulation. Consistent with these findings, our functional work showed that apical AMD RPE EVs induce disease phenotype in the recipient RPE cells, including prominent ultrastructural changes, such as cytoskeletal disruption, vacuolization, melanin degradation and nucleus malformation, alongside increased protein expression of cytoprotective markers, SOD2 and alpha B crystallin; all features resembling phenotypic alterations in AMD RPE. The exposure of control retinal organoids to apical high‐risk EVs led to increased protein expression of specific stress markers, including alpha B crystallin, structural damage to the photoreceptors containing neuroepithelium, and changes suggestive of glial cell activation following injury. We showed that basal and, to a lesser extent, apical AMD RPE EVs induce protein amyloid β‐sheet formation, providing an indication of their contribution to drusen and pseudodrusen biogenesis. We also demonstrated that high‐risk RPE EVs constitute angiogenic stimuli to endothelial colony forming cells for vessel sprouting in a dose‐response manner. Finally, we showed that apical high‐risk RPE EVs are stimulatory to peripheral myeloid cells, suggestive of their involvement in initiation of inflammation. Our study identified previously unrecognised events in active RPE EV signalling that may mediate tissue damage responses in the AMD outer retina.

Oxidative stress is an inherent feature of the RPE metabolism and plays an essential role in normal cellular function and signal transduction. RPE cells are subjected to high levels of physiologic reactive oxygen species (ROS) due to their high metabolic demand and by being exposed to the high levels of oxygen from the choroidal blood flow. Autophagy is a major protective mechanism regulated by a variety of stress stimuli, including oxidative and mitochondrial damage (Kroemer et al., 2010), which leads to the disposal of unwanted proteins and organelles via the lysosomal degradation. Deficiency of autophagic activity leads to the accumulation of inclusion bodies and is associated with neurodegeneration (Komatsu et al., 2006).

Cumulative evidence suggests that deficient autophagy is a feature of AMD. We and others have shown that the autophagic flux is significantly reduced in AMD patient derived RPE cells (Cerniauskas et al., 2020; Golestaneh et al., 2017). Besides autophagy, EV secretion recently came to light as an important process in maintaining cellular homeostasis, with a clear molecular and functional crosstalk with autophagy pathways (reviewed in (Baixauli et al., 2014; Kalluri & LeBleu, 2020)). Interestingly autophagy induction leads to a blockade of EVs production pathways and release. As previously shown, multivesicular bodies (MVBs) that give rise to exosomes, fuse with autophagic vacuoles upon autophagy activation, hence decreasing the overall EVs secretion (Fader et al., 2008). As the cellular metabolic state appears to be crucial in establishing the balance between secretion and autophagic activity, it is not surprising that our study demonstrated enhanced secretion of EVs in a patient specific AMD RPE cell model. The blockage of autophagy, swollen and dysfunctional lysosomes and ‘spillage’ of lysosomal contents into the extracellular deposits beneath the RPE cells (Cerniauskas et al., 2020), appear to be the metabolic cue for cargo redirection and promoting secretion. Additionally, the increased levels of CD63 tetraspanin in the AMD RPE cells (Cerniauskas et al., 2020) may influence enhanced intraluminal vesicles formation within MVBs (van Niel et al., 2011), that could be further stimulated by the increased levels of intracellular ceramides (Trajkovic et al., 2008) (findings of the current study). Overall, the autophagic deficiencies together with changes promoting EVs biogenesis may provide the direct link to the enhanced secretion of EVs observed in our AMD RPE model.

The AMD iPSC‐RPE model generated in our lab displays reduced number of mitochondria, downregulated gene expression of mitochondrial SOD2 ROS scavenging enzyme (Hallam et al., 2017), lower mitochondrial membrane cardiolipin levels and significant global proteome changes indicating mitochondrial dysfunction and oxidative stress response (current study). There appears to be an intimate relationship between autophagy and mitochondria (Okamoto & Kondo‐Okamoto, 2012). Mitochondria require autophagy for their maintenance and mitochondrial function seems to be a critical regulator of autophagy, as mitochondrial respiratory deficiency suppresses autophagic flux and autophagy gene induction in a yeast model (Graef & Nunnari, 2011; Graef & Nunnari, 2011). It is therefore conceivable, that in aged AMD RPE, physiologically challenged by the daily task of photoreceptor outer segments phagocytosis, there is a vicious loop generated by dysfunctional autophagy and mitochondrial deficiency, contributing to the burden of lower cellular energy levels, increased oxidative stress and protein handling defect. The cellular efforts to alleviate the stress conditions may be through increasing the rate of secretion of unwanted and toxic material.

Accordingly, our study shows that AMD RPE EVs contents differs from the control RPE EVs. The transcriptomic analysis of EVs RNA cargo revealed that in AMD RPE, EVs released apically are enriched in molecules involved in integrin signalling, NRF2 mediated oxidative stress response, protein ubiquitination pathway, actin cytoskeleton signalling and regulation of cellular mechanics by calpain protease. Cell stress signalling pathways were also evident in the basal EVs transcriptome, with ERK5 pathway, activated in response to growth factors and stressors, NRF2 mediated oxidative stress response, superoxide degradation and senescence being examples of the significantly overrepresented pathways. As EVs main role is intercellular communication (Kalluri & LeBleu, 2020), based on our data we believe that there is a clear molecular message carried by the AMD RPE EVs to the recipient cells, which involves cell stress (oxidative, protein and senescence).

Our gene enrichment analysis revealed the main signalling nodes in the network of significantly enriched pathways, and this strongly implied angiogenesis signalling via VEGFA in the AMD RPE apical EVs that was inferred to be activated. VEGF is the most potent and primary angiogenic growth factor, necessary for normal vascular development, but also driving pathological new vessel growth (Kim & Byzova, 2014). The current study provides evidence that there is a strong pro‐angiogenic signalling through the RPE cell secretome, reflected in the genetic and protein contents of EVs, and their functional biological role in enhancing angiogenic sprouts formation, which has clear clinical relevance, as our RPE cell lines were derived from patients with the neovascular form of AMD. Our finding of significant apical pro‐angiogenic signalling corresponds well with previous observations of preferentially apical VEGF release in polarised ARPE19 cells (Kannan et al., 2006), and suggests a mechanism that may be a driving force for abnormal blood vessel encroachment into the subretinal space from the choroid as well as stimulation of intraretinal neovascularisation as seen in retinal angiomatous proliferation (Yannuzzi et al., 2001).

The differential lipidomics results of RPE cells suggest significant upregulation of sphingolipids levels and downregulations of glycerolipids and glycerophospholipids including cardiolipins. Sphingolipids, such as ceramides and gangliosides, are ‘bioactive lipids’ and are involved in stress response pathways including apoptosis, autophagy and inflammation (Young et al., 2013). Ceramides are also important for the biogenesis of MVB intraluminal vesicles and exosome secretion (Trajkovic et al., 2008). The increased levels of ceramide in the AMD RPE cells might therefore be one of the reasons for the expanded endolysosomal compartments, as also implicated in a Stargardt disease mouse model pathology (Kaur et al., 2018), and enhanced secretion of EVs. In this study, both apical and basal high‐risk RPE EVs were enriched in GM3 gangliosides and neutral lipids. GM3 stimulates aging and neurodegeneration linked Aβ and α‐synuclein fibril formation, and both types of pathological inclusions are found in the aging retina and/or drusen (Leger et al., 2011; Yamamoto et al., 2005). Here we showed that basal and, to a lesser extent, apical AMD RPE EVs strongly induce aggregation of α‐synuclein, generating amyloid fibrils of similar morphology to those observed in sub‐RPE deposits in human specimen (Isas et al., 2010). Amyloid fibrils are considered to be important for drusen assembly and growth by providing the structural support and platform for binding of other protein and lipid components. Our work therefore suggests that RPE EVs may have an active role in amyloid generation and drusen biogenesis. The protein contents of the high‐risk EVs further supports this possibility. Known drusen components such as clusterin, vimentin, collagen, S100‐A8 and fibronectin, were found to be enriched specifically in high risk EVs. The protein and lipid contents of the EVs might therefore provide a scaffold for sub‐RPE debris accumulation and drusen growth.

It is established that EVs are potent transmitters of molecular information between cells and body organs (Mathieu et al., 2019). In this work we show that RPE cells internalise exogenous vesicles, and the transferred cargo influences the recipient cells metabolism. Apical AMD RPE EVs exert a remarkable pathological effect on control RPE, inducing cytoplasmic vacuolisation, cytoskeletal disruption, melanin degradation, changes to the nucleus and condensation of the cytoplasm. These cellular ultrastructural changes accompany significantly decreased rate of cathepsin D processing, manifested by 50% lower levels of pro‐cathepsin D and 30% lower levels of mature heavy chain of cathepsin D, one of the major lysosomal proteases, and several fold increased levels of stress response proteins, SOD2 and alpha B crystallin. The upregulation of SOD2 is likely suggestive of oxidative stress response in the recipient cells, whereas alpha B crystallin is a molecular chaperone with a wide range of cytoprotective roles, including anti‐oxidative and anti‐apoptotic (Kannan et al., 2016). Interestingly, alpha B crystallin was found to be specifically overexpressed in wet and dry AMD RPE macular specimens and was proposed to be a biomarker for AMD (De et al., 2007). It has previously been shown that alpha B crystallin is secreted by RPE in association with exosomes, preferentially towards the apical side and exerts anti‐apoptotic function upon oxidative stress by suppressing caspase 3 and PARP cleavage (Sreekumar et al., 2010). Correspondingly, in our study, despite pronounced ultrastructural changes in the recipient RPE cells, we did not observe caspase 3 nor PARP cleavage; however, downregulation of pro‐caspase 3 and a trend towards upregulation of PARP was evident. This might suggest pro‐survival mechanisms, leading to accumulation of stressed and phenotypically damaged RPE cells, which act as disease messengers for both photoreceptors and other adjacent healthy RPE cells.

Our study showed evidence for calpain mediated vimentin cleavage and cytoskeleton disruption in the recipient RPE due to the uptake of apical AMD RPE EVs. These events are consistent with pyroptotic cell death inherently associated with inflammation (Davis et al., 2019). Inflammasome activation in macrophages exposed to AMD drusen has been demonstrated, and its protective role in neovascularization development has been suggested by suppressing the RPE secretion of VEGF (Doyle et al., 2012). Another study has elegantly shown that necrotic pathways may be responsible for RPE cell death in AMD, and these are associated with macrophage infiltration and activation in the outer retina (Murakami et al., 2014). Our study identified that apical AMD RPE EVs initiate a pro‐inflammatory response in human THP‐1 macrophages. This is likely mediated by the EVs cargo, for example molecules involved in oxidative stress signalling and lipids GM3 gangliosides that have been shown to mediate oxidative stress induced toxicity (Sohn et al., 2006). Given that cell necrosis can activate inflammasome assembly in immune cells through the release of danger‐associated molecular patterns (DAMPs) (Iyer et al., 2009), it might be that AMD RPE EVs constitute a form of DAMPs and are involved in innate immune response activation. Infiltration of macrophages has been observed in AMD regressing drusen, that is thought to be a correlate of RPE cell death (Sarks et al., 1999), and in the light of the above findings, it likely represents an orchestrated response to tissue injury, which might involve RPE EV signalling.

Our study shows that retinal organoids containing photoreceptors treated with both apical and basal high‐risk RPE EVs, exhibit signs of cell stress and activated compensatory responses, such as altered mitochondrial dynamics towards the elongation of mitochondria, increased expression of cytoprotective alpha B crystallin and SOD2, and increased expression of cathepsin D. We hypothesise that the exogenous EVs uptake by Müller glia induces neuroprotective effects of alpha B crystallin and the activation of Müller glia regenerative potential, as demonstrated by the presence of cells positive for neural progenitor Pax6, retinal progenitor Vsx2 and alpha B crystallin (Ahmad et al., 2011; Hampton et al., 2020). Alpha B crystallin has been recognised to have a neuroprotective role through activation of glial cells in models of neurodegeneration (Bsibsi et al., 2013; Hampton et al., 2020) and our study is consistent with these observations. Our findings therefore suggest that the apical high‐risk RPE EVs may cause injury to retinal cells and constitute a stimulus for glial cells activation in attempt to restore tissue homeostasis. This may be a trigger for glia migration to the subretinal locations, chronic inflammation and angiogenesis, contributing to the photoreceptor cells injury and loss during the course of the disease (Vecino et al., 2016).

In conclusion, our work demonstrated that AMD RPE EVs are potent messengers of disease phenotype to both, RPE and photoreceptors. Further understanding of the biology of EVs is warranted to evaluate their potential as biomarkers or treatment strategies for AMD.

## AUTHOR CONTRIBUTIONS

Marzena Kurzawa‐Akanbi: Conceptualization; Data curation; Formal analysis; Funding acquisition; Investigation; Methodology; Project administration; Supervision; Visualization; Writing – original draft; Writing – review & editing. Phillip Whitfield: Data curation; Formal analysis; Funding acquisition; Methodology; Software; Writing – review & editing. Florence Burté: Formal analysis; Methodology; Software; Visualization; Writing – review & editing. Pietro Maria Bertelli: Data curation; Formal analysis; Investigation; Methodology; Visualization; Writing – review & editing. Varun Pathak: Data curation; Formal analysis; Investigation; Methodology; Visualization; Writing – review & editing. Mary Doherty: Data curation; Formal analysis; Investigation; Methodology; Software; Writing – review & editing. Birthe Hilgen: Data curation; Formal analysis; Methodology; Software; Visualization; Writing – review & editing. Mark Platt: Data curation; Methodology; Writing – review & editing. Rachel Queen: Formal analysis; Methodology; Software; Visualization; Writing – review & editing. Jonathan Coxhead: Data curation; Methodology; Writing – review & editing. Andrew Porter: Data curation; Writing – review & editing. Maria Öberg: Data curation; Formal analysis; Visualization; Writing – review & editing. Daniela Fabrikova: Data curation; Methodology; Writing – review & editing. Tracey Davey: Data curation; Methodology; Writing – review & editing. Chia Shyan Beh: Formal analysis; Visualization; Writing – review & editing. Maria Georgiou: Data curation; Formal analysis; Methodology; Visualization; Writing – review & editing. Joseph Collin: Data curation; Formal analysis; Methodology; Visualization; Writing – review & editing. Veronika Boczonadi: Data curation; Formal analysis; Methodology; Visualization; Writing – review & editing. Anetta Härtlova: Investigation; Methodology; Supervision; Writing – review & editing. Michael Taggart: Funding acquisition; Supervision; Writing – review & editing. Jumana Al‐Aama: Funding acquisition; Writing – review & editing. Jasenka Guduric‐Fuchs: Investigation; Methodology; Supervision; Writing – review & editing. Lyle Armstrong: Conceptualization; Funding acquisition; Writing – review & editing. Majlinda Lako: Conceptualization; Formal analysis; Funding acquisition; Investigation; Methodology; Project administration; Resources; Software; Supervision; Visualization; Writing – original draft; Writing – review & editing. All authors have read and approved the final version of the manuscript.

## CONFLICTS OF INTEREST

The authors declare no relevant conflict of interest.

## Supporting information

Supporting Information

Supporting Information

## Data Availability

Data supporting this manuscript is available in the Supplementary Materials. The mass spectrometry proteomics data were deposited to the ProteomeXchange Consortium via the MassIVE partner repository with the dataset identifiers: MassIVE: MSV000088713 and Proteome Xchange: PXD031135. https://eur03.safelinks.protection.outlook.com/ https://3A/2F/2Fmassive.ucsd.edu/2F%26data=04/7C01/7Cmarzena.kurzawa2/40newcastle.ac.uk/7Cadac61a8631d4c67021608d9dcfea252/7C9c5012c9b61644c2a91766814fbe3e87/7C1/7C0/7C637783808461947992/7CUnknown/7CTWFpbGZsb3d8eyJWIjoiMC4wLjAwMDAiLCJQIjoiV2luMzIiLCJBTiI6Ik1haWwiLCJXVCI6Mn0/3D/7C3000%26sdata=XfvCr8AqqItkdoIHCBsLxYEYXprKixC0t9oBqT9DEV8/3D%26reserved=0 The EVs transriptomics data were submitted to the GEO repository with the dataset identifier GSE190263. https://eur03.safelinks.protection.outlook.com/ https://3A/2F/2Fwww.ncbi.nlm.nih.gov/2Fgeo/2Fquery/2Facc.cgi/3Facc/3DGSE190263%26data=04/7C01/7Cmarzena.kurzawa2/40newcastle.ac.uk/7Ce6ab7683eb784d7375d608d9dce08ea7/7C9c5012c9b61644c2a91766814fbe3e87/7C1/7C0/7C637783679278725193/7CUnknown/7CTWFpbGZsb3d8eyJWIjoiMC4wLjAwMDAiLCJQIjoiV2luMzIiLCJBTiI6Ik1haWwiLCJXVCI6Mn0/3D/7C3000%26sdata=Db17MMe03zc9OeIdZXNiRBwDNoedT2E4iMcsdoR7zFo/3D%26reserved=0
