# Supplementary material for: Retinal pigment epithelium extracellular vesicles are potent inducers of age‐related macular degeneration disease phenotype in the outer retina
Source: J Extracell Vesicles. 2022 Dec 21;11(12):12295. doi: 10.1002/jev2.12295 (PMC9772497; doi:10.1002/jev2.12295)
Supplement: Supplementary file 1 — Supporting Information [file JEV2-11-12295-s001.docx]

**Supplementary Figures**

**Retinal pigment epithelium extracellular vesicles are potent inducers of age-related macular degeneration disease phenotype in the outer retina**

Marzena Kurzawa-Akanbi^1#^, Phillip Whitfield^2^, Florence Burté^1^, Pietro Maria Bertelli^3^, Varun Pathak^3^, Mary Doherty^4^, Birthe Hilgen^1^, Lina Gliaudelytė^5^, Mark Platt^6^, Rachel Queen^1^, Jonathan Coxhead^1^, Andrew Porter^1^, Maria Öberg^7,8^, Daniela Fabrikova^7,8^, Tracey Davey^9^, Chia Shyan Beh^1^, Maria Georgiou^1^, Joseph Collin^1^, Veronika Boczonadi^1^, Anetta Härtlova^7,8^, Michael Taggart^1^, Jumana Al-Aama^10^, Viktor Korolchuk^1^, Christopher M Morris^5^, Jasenka Guduric-Fuchs^3^, David Steel^1^, Reinhold Medina^3^, Lyle Armstrong^1^ and Majlinda Lako^1#^.

1. Biosciences Institute, Faculty of Medical Sciences, Newcastle University, Newcastle upon Tyne, UK.
2. Glasgow Polyomics, College of Medical, Veterinary and Life Sciences, University of Glasgow, Glasgow, UK.
3. The Welcome-Wolfson Institute for Experimental Medicine, Queen’s University Belfast, Belfast, UK.
4. Lipidomics Research Facility, University of the Highlands and Islands, Inverness, UK.
5. Translational and Clinical Research Institute, Newcastle University, Newcastle upon Tyne, UK.
6. Loughborough University, Loughborough, UK.
7. Institute of Biomedicine, Department of Microbiology and Immunology, Sahlgrenska Academy, University of Gothenburg, Gothenburg, Sweden.
8. Wallenberg Center for Molecular and Translational Medicine, University of Gothenburg, Gothenburg, Sweden.
9. Electron Microscopy Research Services, Newcastle University, Newcastle upon Tyne, UK.
10. Faculty of Medicine, King Abdulaziz University, Saudi Arabia.

*# to whom the correspondence should be addressed:*

Prof. Majlinda Lako and Dr. Marzena Kurzawa-Akanbi

Newcastle University

Biosciences Institute

International Centre for Life

Central Parkway

Newcastle upon Tyne

NE1 3BZ

Email: majlinda.lako@ncl.ac.uk and marzena.kurzawa2@ncl.ac.uk

**
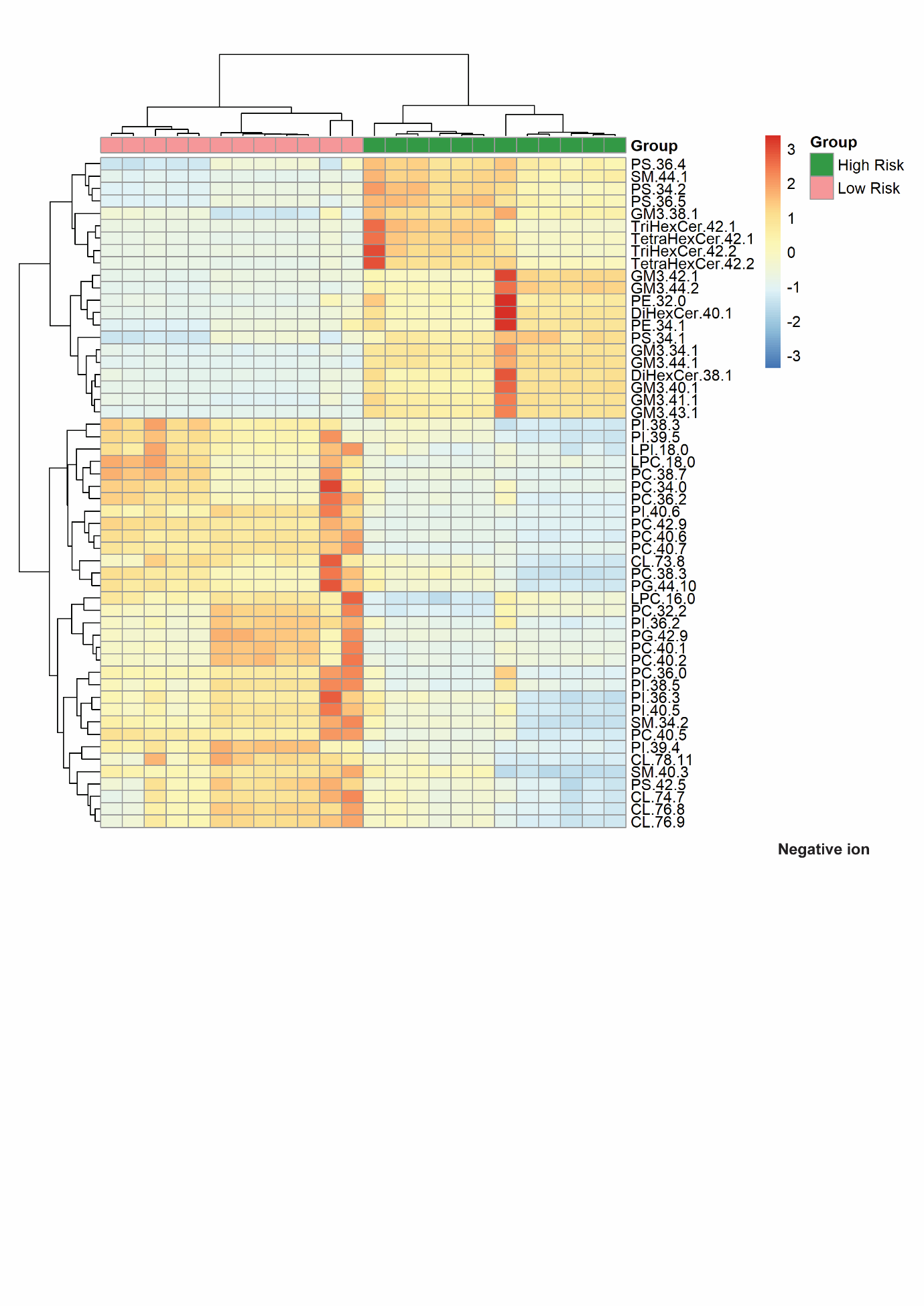
**

**Supplementary Figure 1:** **Lipidomics analysis of low and high-risk RPE cell lines.** Heatmap shows supervised clustering of statistically significantly changed lipid species in high-risk versus low-risk RPE, based on t-test analysis and p value <0.0001 cut-off, from negative ion mode mass spectrometry analysis.

**
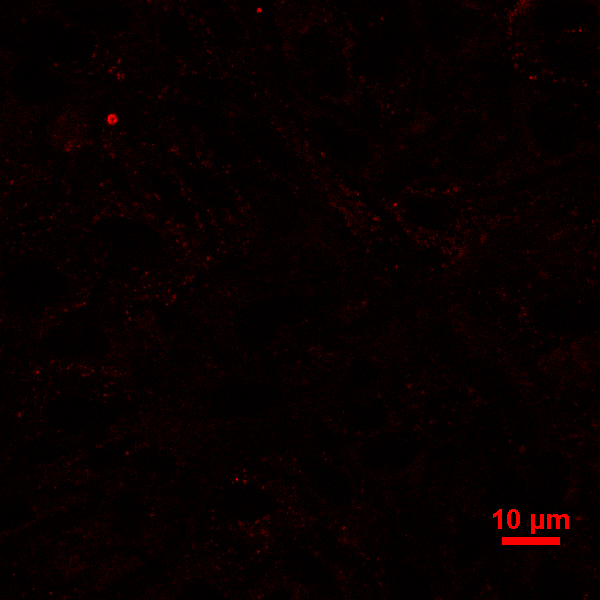
**

**Supplementary Figure 2: Negative control for EV fluorescent labelling, purification and *in vitro* EV RPE uptake study.** Control reaction was carried out in parallel to EV samples, with DPBS used instead of EV preparations. Representative image taken 24 hours post-exposure of the control sample to RPE cells, as indicated in the Methods section, is shown.


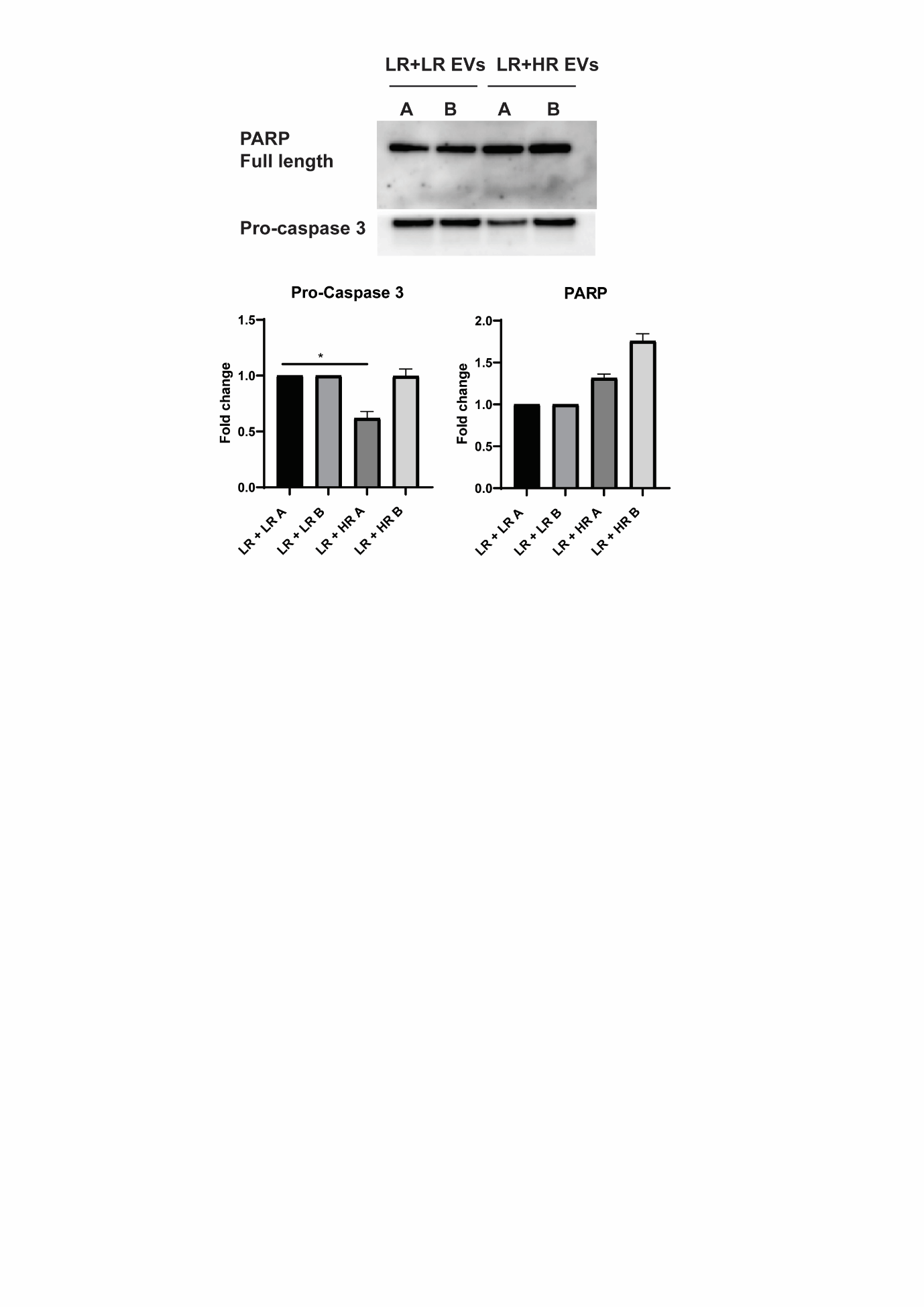


**Supplementary Figure 3: Western blot analysis of apoptosis markers, PARP and caspase 3 in low-risk RPE cells exposed to exogenous RPE EVs.** No cleavage fragments of PARP nor pro-caspase 3 were detected. Decreased amounts of pro-caspase 3 in low-risk RPE treated with high-risk EVs were evident and a trend towards upregulation of PARP in low-risk cells treated with both apical and basal high-risk EVs was observed, likely indicating genotoxic stress and activation of pro-survival mechanisms.


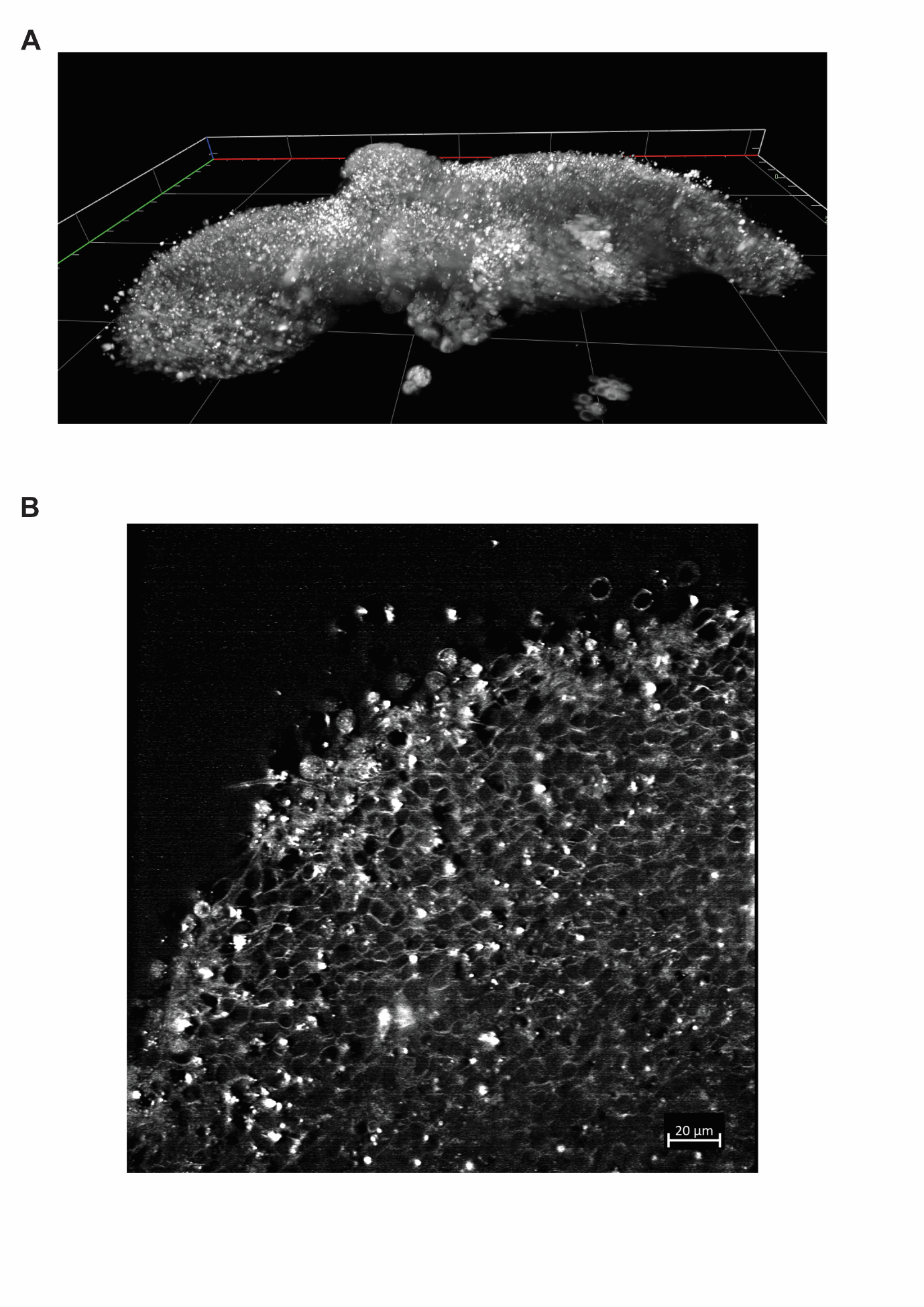


**Supplementary Figure 4: RPE EVs uptake by retinal organoids.** EVs were fluorescently labeled with ExoGlow-Membrane EV Labeling Kit (SBI) and exposed to mature retinal organoids (differentiation day 217) for 24 hours. Live cell imaging was performed using a Zeiss Lattice Lightsheet 7 fluorescence microscope. (A) Three-dimensional image of the organoid structure showing exogenous EV-derived fluorescent staining of various retinal cell types. The grid scale bar 200 µm × 200 µm. (B) Single focal plane image demonstrating EV-derived fluorescent staining of the apical layer of the retinal organoid including a putative photoreceptor cell layer. Membrane and cytoplasmic staining are visible. Scale bar 20 µm.


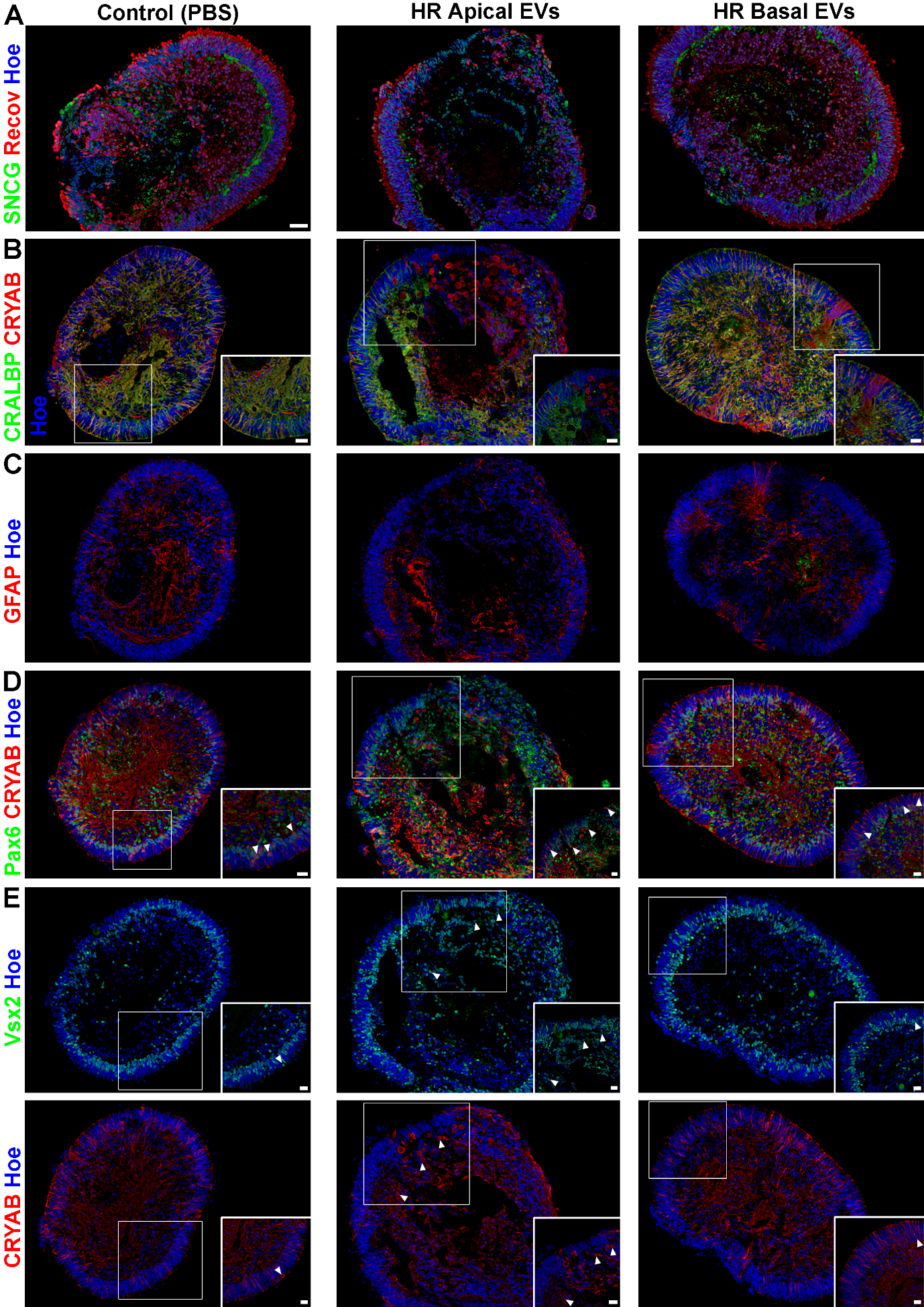


**Supplementary Figure 5: Retinal organoid exposure to high-risk apical and basal EVs.** Control retinal organoids were treated with high-risk apical/basal EVs in 6 doses across two weeks. ICC analysis was performed, with thin organoid sections stained for (A) photoreceptor recoverin (Recov) and ganglion cell gamma-synuclein (SCNG) markers; (B) Müller glia cellular retinaldehyde binding protein (CRALBP) marker and alpha B crystallin (CRYAB); (C) astrocytes glial fibrillary acidic protein (GFAP) marker; (D) neural progenitor cells Pax6 marker and CRYAB (arrowheads denote cells immunopositive for alpha B crystallin and Pax6 protein expression); (E) proliferative retinal progenitor cells visual system homeobox 2, Vsx2, marker and CRYAB (arrowheads denote cells positive for both Vsx2 and CRYAB staining). Cell nuclei were counterstained with Hoechst. Scale bars: A – 50 µm; insets – 20 µm.


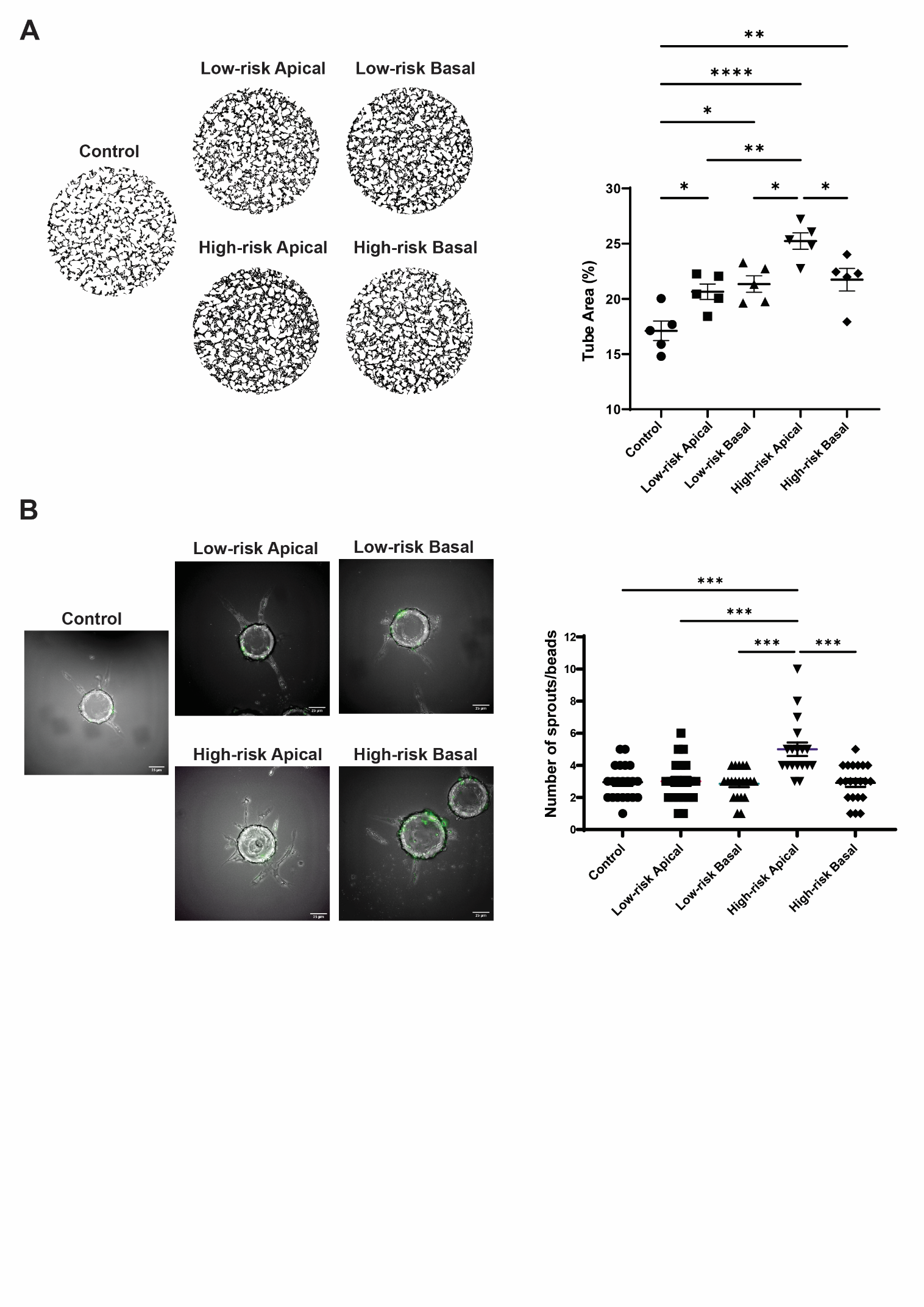


**Supplementary Figure 6: High-risk apical AMD RPE EVs enhance endothelial colony forming cells (ECFCs) 3D tube formation and angiogenic sprouting.** (A) ECFCs were treated with low-risk and high-risk apical and basal RPE EVs for 18 hours, followed by ECFCs embedding in 3D Matrigel. Images were taken at 48 hours of cells being embedded in Matrigel and ECFC tube area (percentage per well) was calculated. N = 5 wells per experimental group. 4 different ECFCs clones were tested, and representative data is shown. Statistical analysis of ECFCs tube area was performed using one-way ANOVA followed by Tukey’s multiple comparisons tests. (B) ECFCs attached to beads were treated with low-risk and high-risk apical and basal RPE EVs for 18 hours, followed by embedding in Fibrin. Images were taken at day 5 post-embedding. Scale bars 25 µm. Number of sprouts per bead was calculated. N ≥ 18 beads per experimental group. 3 different ECFCs clones were tested with representative data shown. Statistical analysis was performed using one-way ANOVA followed by Dunn’s multiple comparisons test. * p <0.05, ** p <0.01, *** p <0.001, **** p <0.0001. Mean ± SEM shown.


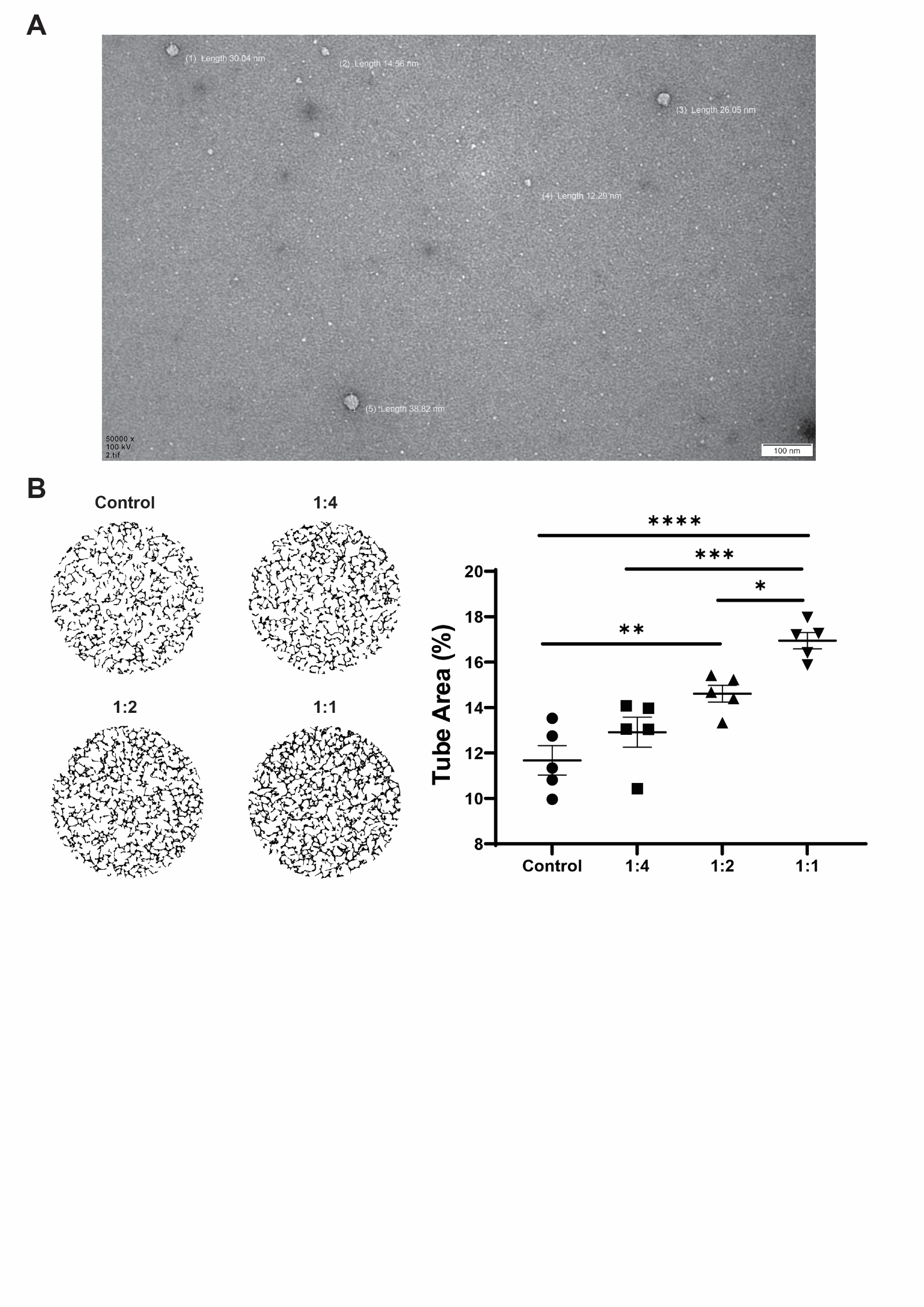


**Supplementary Figure 7: Dose-response experiments to evaluate the effective contribution of high-risk apical EVs amounts to their pro-angiogenic properties.** (A) Representative TEM image of high-risk apical EVs tested in the ECFCs 3D tube formation assays. Scale bar 100 nm. (B) Different ratios of RPE EVs to ECFCs were tested (based on cell counts ratios to ensure physiological EV amounts). Statistically significant increase in ECFCs tube area compared to the assay control (PBS) was observed for 1:2 and 1:1 ratio, indicating the stimulatory effect of the EVs contents that was further enhanced by the increased EVs amounts. Statistical analysis of ECFCs tube area was performed using normality tests and one-way ANOVA followed by Tukey’s multiple comparisons tests. * p <0.05, ** p <0.01, *** p <0.001, **** p <0.0001. Mean ± SEM shown.


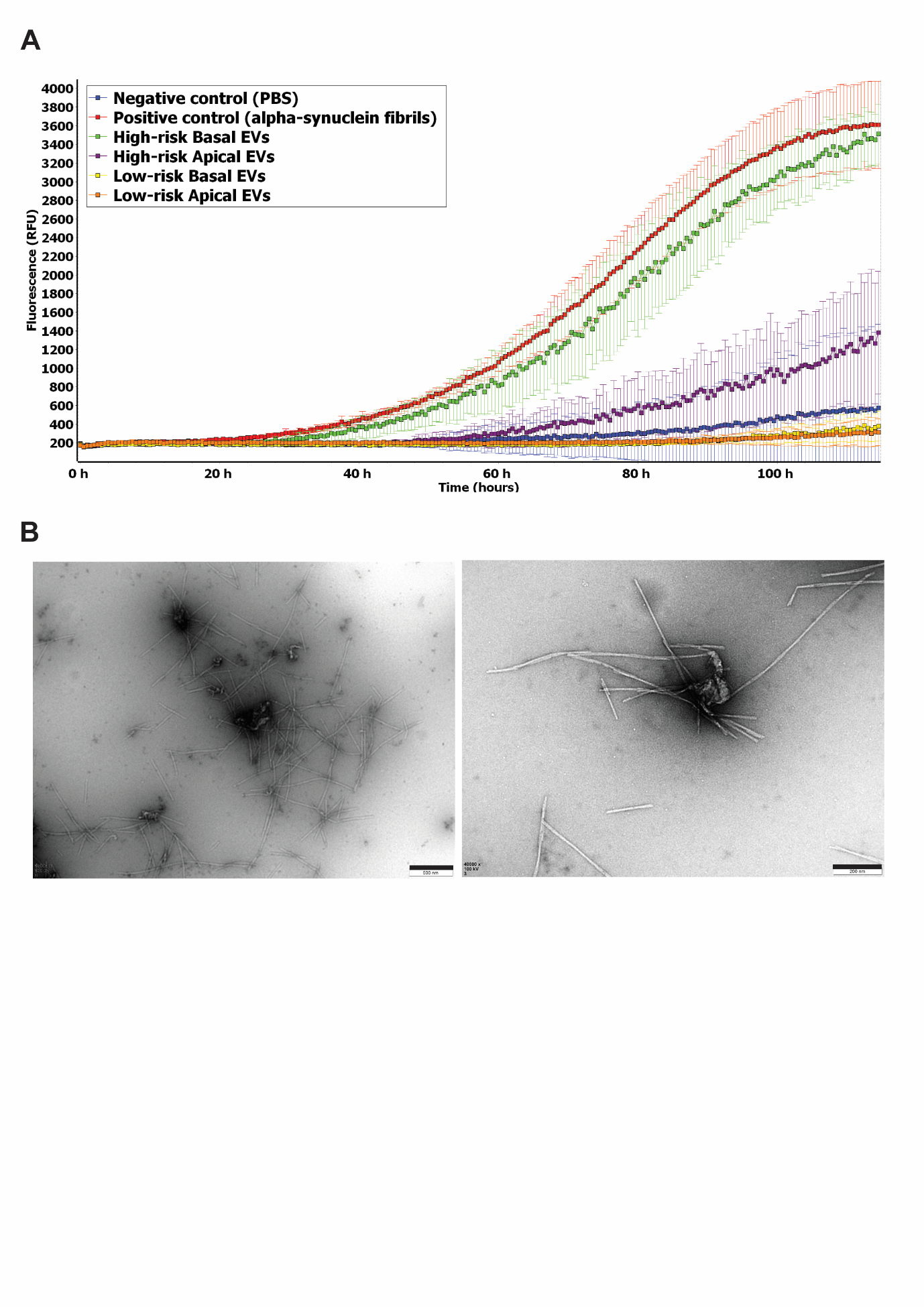


**Supplementary Figure 8: Basal high-risk RPE EVs stimulate protein aggregation and amyloid fibril formation.** α-synuclein aggregate formation has been used to verify RPE EVs-induced amyloid formation and potential contribution to drusen and subretinal drusenoid deposits (pseudodrusen) formation. (A) Exponential curves indicate the generation of α-synuclein fibrils (Mean ± standard deviation of 3 replicates presented). In contrast to low-risk RPE EVs, high-risk basal RPE-EVs are potent inducers of α-synuclein fibril formation, with a smaller effect on amyloid formation from the apical high-risk RPE-EVs. (B) Transmission electron microscopy images of generated α-synuclein fibrils in the presence of basal high-risk RPE EVs. Intact and disrupted EVs are present in the amyloid fibril net, suggestive of their contribution to fibril generation. Scale bars – 500 nm (left) and 200 nm (right).


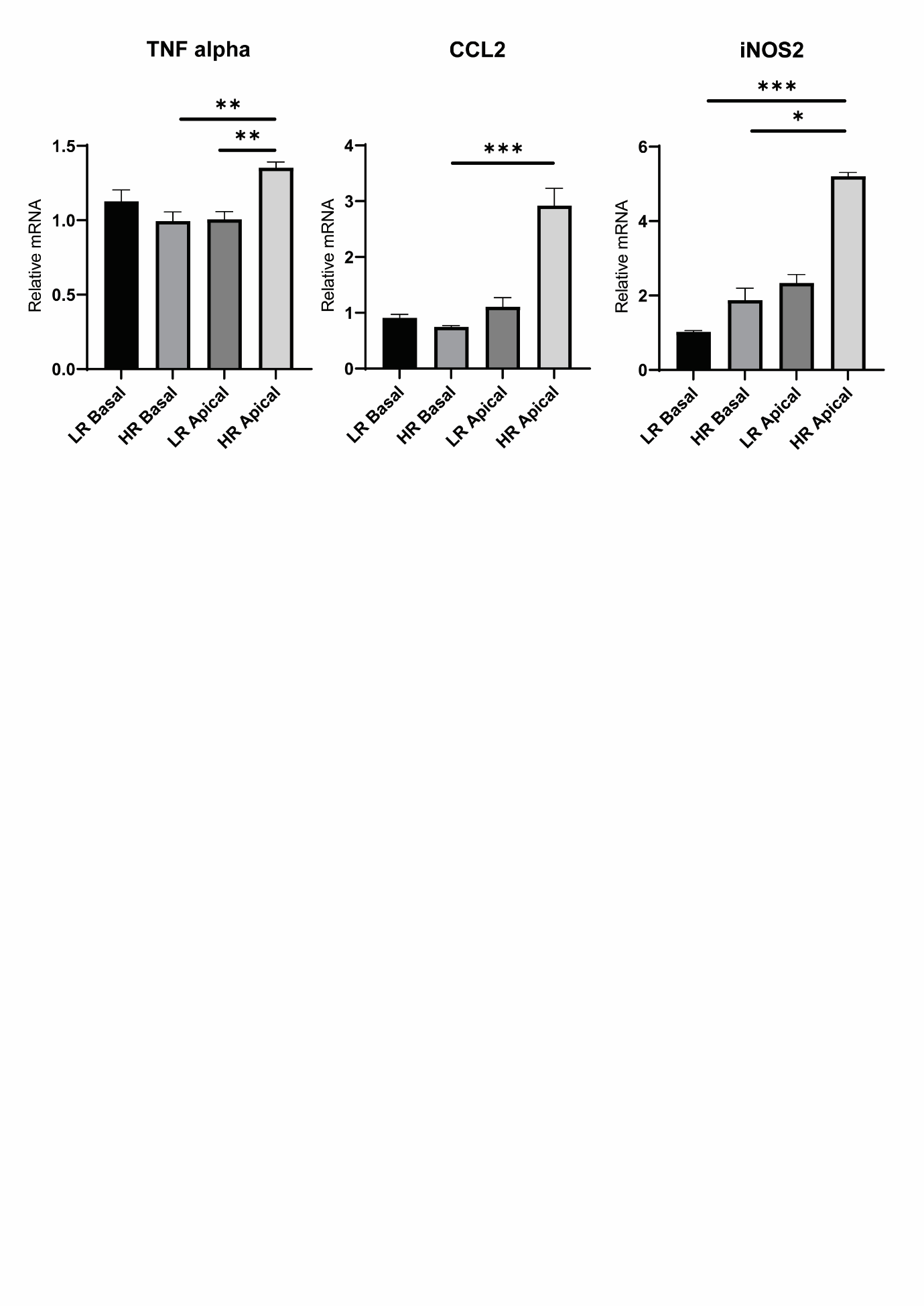


**Supplementary Figure 9: Immuno-modulatory potential of RPE EVs**. Apical high-risk EVs induced enhanced gene expression of inflammatory genes in THP-1 derived macrophages. Mean ± SEM presented, n=6. Data was analysed for normality by Shapiro-Wilk test, followed by ANOVA and either Tukey’s (parametric) or Dunn’s (non-parametric) multiple comparisons tests. * p <0.05, ** p <0.01, *** p <0.001.
